# Inhibition of DNA Methylation Alters Chromatin Organization, Nuclear Positioning and Activity of 45S rDNA Loci in Cycling Cells of *Q. robur*

**DOI:** 10.1371/journal.pone.0103954

**Published:** 2014-08-05

**Authors:** Vedrana Vičić Bočkor, Darko Barišić, Tomislav Horvat, Željka Maglica, Aleksandar Vojta, Vlatka Zoldoš

**Affiliations:** 1 Faculty of Science, University of Zagreb, Department of Molecular Biology, Zagreb, Croatia; 2 Friedrich Miescher Institute for Biomedical Research, Basel, Switzerland; 3 Ecole Polytechnique Fédéral de Lausanne, Lausanne, Switzerland; Ludwig-Maximilians-Universität München, Germany

## Abstract

Around 2200 copies of genes encoding ribosomal RNA (rRNA) in pedunculate oak, *Quercus robur*, are organized into two rDNA loci, the major (NOR-1) and the minor (NOR-2) locus. We present the first cytogenetic evidence indicating that the NOR-1 represents the active nucleolar organizer responsible for rRNA synthesis, while the NOR-2 probably stays transcriptionally silent and does not participate in the formation of the nucleolus in *Q. robur*, which is a situation resembling the well-known phenomenon of nucleolar dominance. rDNA chromatin topology analyses in cycling root tip cells by light and electron microscopy revealed the minor locus to be highly condensed and located away from the nucleolus, while the major locus was consistently associated with the nucleolus and often exhibited different levels of condensation. In addition, silver precipitation was confined exclusively to the NOR-1 locus. Also, NOR-2 was highly methylated at cytosines and rDNA chromatin was marked with histone modifications characteristic for repressive state. After treatment of the root cells with the methylation inhibitor 5-aza-2′-deoxycytidine, we observed an increase in the total level of rRNA transcripts and a decrease in DNA methylation level at the NOR-2 locus. Also, NOR-2 sites relocalized with respect to the nuclear periphery/nucleolus, however, the relocation did not affect the contribution of this locus to nucleolar formation, nor did it affect rDNA chromatin decondensation, strongly suggesting that NOR-2 has lost the function of rRNA synthesis and nucleolar organization.

## Introduction

In plants, NOR (nucleolar organizer region) locus is composed of multiple repeats of ribosomal (rRNA) genes that encode the 45S pre-ribosomal transcript, which is processed to generate mature 18S, 5.8S and 26–28S rRNA molecules. The redundancy of the information allows for a fraction of rRNA genes within a locus to be transcriptionally active while others stay silent, and the proportion of silent and active genes is determined by cellular needs for protein synthesis [1]. The current model of rDNA chromatin organization proposes that the subset of silent rRNA genes stays highly condensed (heterochromatinized), and the subset of rRNA genes which that are actively transcribed are contained in a more decondensed rDNA chromatin domain [2], [3]. Therefore, there is a clear association between the rDNA chromatin state, transcriptional activity of rRNA genes and the plasticity of rDNA chromatin, i.e. the possibility for silent rRNA genes to be reactivated by an epigenetic on/off switch [3], [4].

Epigenetic mechanisms regulating the transcriptional state of rRNA genes associated with rDNA chromatin organization have been well studied in plant hybrid species exhibiting a phenomenon known as nucleolar dominance. In these hybrids, rDNA loci of only one parent are transcriptionally active while rDNA loci of the other parent stay silent [5], [6]. Differential activity of rRNA genes in plant nucleolar dominance is regulated by chromatin-mediated changes, which involve specific histone modifications in partnership with DNA methylation [3], [7]. In *Arabidopsis*, the “off” switch in rRNA gene regulation involves cytosine methylation, histone deacetylation, H3K9 dimethylation (H3K9me2) and chromatin condensation, while the “on” switch involves loss of cytosine methylation, histone H3 and H4 hyperacetylation, H3K4 trimethylation (H3K4me3) and chromatin decondensation [8]. Silent rRNA genes can be reactivated by the use of inhibitors of DNA methylases and histone deacetylases, further confirming the direct involvement of these epigenetic mechanisms in the transcriptional regulation of rRNA genes [7], [9], [10]. Both CpG methylation by MET1 (*Methyltransferase 1*) and siRNA-guided DRM2 *(Domains Rearranged Methyltransferase 2)* directed DNA methylation followed by histone deacetylation are involved in rRNA gene silencing in hybrid *A. suecica* [3], [11]. DRM2 is responsible for asymmetric CNN (where N is any nucleotide except G) methylation, which is a signal for binding of *Methyl Binding Domain* MBD1 and MBD6 proteins and further recruitment of *Histone Deacethylases* (HDACs) needed to silence rRNA genes.

Active rRNA genes associate with silver-reducing argyrophylic proteins and can be visualized at the cytogenetic level by a simple staining technique [12]–[14]. Therefore, Ag-staining has become a tool for identification and localization of actively transcribed rDNA loci. On the other hand, the organization pattern (i.e. topology) of rDNA chromatin within a certain locus can be an indirect evidence of its activity. Observations from many studies suggested that transcriptionally silent NORs contain highly condensed rDNA chromatin during the entire cell cycle. By contrast, transcriptionally active NORs contain condensed small rDNA knobs interconnected by dispersed (more decondensed) rDNA chromatin [15]–[21]. Indeed, by combining FISH (fluorescent *in situ* hybridization) and S1 nuclease protection data, it has been shown that partial rDNA chromatin decondensation is a cytogenetic manifestation of active rRNA gene transcription of a given locus [18]. Also, thin secondary constrictions (SC) visible on (pro)metaphase chromosomes result from reduced condensation of rDNA chromatin and probably from transcriptional activity of rRNA genes [22], [23].

The position of competent, i.e. transcriptionally active, rDNA locus/loci determines the position of the nucleolus/nucleoli. This was initially observed by Heitz and McClintock in the early cytogenetic era [24], [25] and later confirmed by a large number of studies. Indeed, the integrity of the nucleolus depends on the expression of rRNA genes [26]–[31], and the tendency of NOR-bearing chromosomes to associate with nucleolus/nucleoli correlates with the transcriptional competency of NORs. Therefore, the association of a certain rDNA locus with a nucleolus is an indication of transcriptional activity of rRNA genes within this locus, as actively transcribed rRNA genes are an integral part of the nucleolus [11], [32], [33]. In triticale, a wheat-rye hybrid, transcriptionally active NORs of wheat origin are found associated with the nucleoli while silent NORs of rye origin are located proximal to the nucleoli [20]. Also, it has been shown in HeLa and LEP cells that large-scale positioning of NOR-bearing chromosomes with regard to nucleoli is linked to the transcriptional activity of rRNA genes [34].

In pedunculate oak, *Quercus robur*, there are around 2200 copies of rRNA genes organized as two rDNA loci – the major (NOR-1) and the minor locus (NOR-2) – and maximally two nucleoli, with a high frequency of fusing into a single large nucleolus, are present in the nuclei of root tip cycling cells [35]. In the presented work we aimed to deduce the transcriptional status of these two NORs by studying: (1) their localization relative to the nucleolus; (2) their chromatin topology (i.e. rDNA chromatin organization); (3) staining with silver, as well as (4) associated epigenetic modifications and (5) the level of transcriptional activity of rRNA genes before and after the treatment with DNA methylation inhibitor 5-aza-2′deoxycytidine (5-aza-2′-dC). The obtained results suggest that only the NOR-1 locus is transcriptionally active and participates in the formation of one nucleolus or rarely two nucleoli (formed by two NOR-1 sites). In addition, NOR-1 locus showed cell-stable dimorphism regarding rDNA chromatin organization (highly condensed *versus* decondensed rDNA chromatin state). Therefore, it appears that only a subset of rRNA copies within the NOR-1 locus is transcriptionally active and this subset is not random since the rDNA chromatin topology is mitotically inherited. Although the inhibition of DNA methylation by 5-aza-2′-dC relocated NOR-2 locus closer to the nucleolus/nucleoli, we found no cytological evidence of its transcriptional activity, suggesting that rRNA genes residing within this locus probably exist as pseudogenes.

## Materials and Methods

### Plant material

The acorns of *Q. robur*, L. (*Fagaceae*) used in this study were collected on location Jasterbarsko with the permission of Croatian Forest Research Institute by their employees and given to the authors to grow under laboratory conditions. The authors therefore required no special field permit for obtaining the samples. The species used in this study are not endangered or protected. Both oak acorns and seeds of triticale were germinated on moist cotton in Petri dishes at room temperature. For silver staining, fluorescence *in situ* hybridization (FISH) and immunodetection of 5-methylcytosine (5-mC), root tips were fixed in freshly prepared ethanol-acetic acid (3∶1) overnight at 4°C, transferred to 70% ethanol and stored at −20°C until use. Prior to fixation, oak root tips were treated with 2 µM 8-hydroxiquinoline for 4 h at 18°C. Root tips of triticale were pre-treated with ice-cold water for 24 h and fixed in ethanol-acetic acid (3∶1) overnight at 4°C, transferred to 70% ethanol and stored at −20°C until use. Root tips for immunofluorescence (IF) experiments were fixed in 2% (w/v) paraformaldehyde, 1% (w/v) PVP, 0.5% (v/v) Triton X-100 in 1×PBS for 20 min at room temperature and then washed extensively in 1×PBS (phosphate buffered saline).

### Slide preparation

Oak nuclei suspensions for silver staining, FISH and immunodetection of 5-mC were prepared by incubating root tips in the enzymatic mixture [8% (w/v) cellulase RS, 4% (w/v) hemicellulase, 4% (w/v) pectolyase Y-23 in 0.1 M citrate buffer pH 4.8] for 1.5–3 h at 37°C. Triticale root tips were digested in the same enzymatic mixture for 30 min at 37°C. Root tips were then macerated in a droplet of 60% acetic acid, the obtained macerate was transferred into a 1.5 mL Eppendorf tube and nuclei were collected by centrifugation at 800×g for 3 min. The supernatant was discarded and the pellet was washed twice with freshly prepared fixative. Finally, nuclei were resuspended in an appropriate volume of the fixative and 2–3 µl of the suspension were dropped on clean slides and air-dried. For immunodetection of histones, meristems were digested with the enzymatic mixture [8% (w/v) cellulase RS, 4% (w/v) hemicellulase, 4% (w/v) pectolyase Y-23 in 0.1 M citrate buffer pH 4.8] for 90 min at 37°C, washed in 1×PBS for at least 15 min, transferred into a droplet of dH_2_O and squashed. After freezing, the coverslips were removed and slides transferred to 1×PBS.

### Preparation of ultra-thin sections for electron microscopy

Root tips were fixed in 1% (v/v) glutaraldehyde and 0.25% (v/v) saturated aqueous picric acid solution in phosphate buffer (0.05 M Na_2_HPO_4_, 0.05 M KH_2_PO_4_) for 1 h at room temperature. Material was then dehydrated in a graded ethanol series, embedded in LR White (medium) resin (London Resin Company LTD) and polymerized at 52°C overnight. Ultra-thin sections (0.1 µm) were cut with a glass knife on an ultra-microtome (Reichert OmU3). Sections were picked up on gold 100 mesh grids with a carbon-coated support Formvar film. Grids with sections were incubated overnight at 37°C prior to *in situ* hybridization (ISH).

### Silver staining

Cytogenetic slides for silver staining were prepared by dropping the chromosome suspension onto the ice-cold slides and air-dried. Then, the chromosome spreads were incubated in a silver nitrate solution consisting of 2 parts 50% AgNO_3_ and 1 part 2% gelatin/1% formic acid solution under a plastic coverslip at room temperature for 15 to 30 min. After achieving appropriate coloration, the slides were washed extensively in distilled water and mounted as previously described.

### Treatments with 5-aza-2′-deoxycytidine

5-aza-2′deoxycitidine (Sigma) was dissolved in 10 mM PBS and stored at 4°C and −20°C until use. Acorns were germinated on moist cotton in Petri dishes in a humid place at room temperature. After 7 days of germination, root tips of the seedlings were immersed in a Petri dish containing 50 mL of 10 µM 5-aza-2′-dC and left to grow for 10 days at room temperature. Water solution containing 5-aza-2′-dC was replaced every 2 days keeping the concentration effective. Seedlings for the control experiment were grown in sterile water in the same environmental conditions. Meristematic tissue was isolated from root tips and then fixed as previously described [35].

### Fluorescence *in situ* hybridization

Slides were treated with RNase A solution (50 µg/mL in 2×SSC) for 1 h at 37°C, washed in 2×SSC and treated with Proteinase K (0.1 mg/mL) for 15 min. After washing in 2×SSC, the slides were dehydrated through ethanol series and air-dried. The hybridization mixture consisted of 50% (v/v) formamide, 10% (w/v) dextran sulphate, 10% SDS, 1.5–2 ng/µL Cy3 labelled 18S rDNA probe in 2×SSC. In case of triticale samples, an additional 1.5–2 ng/µL of wheat (*Triticum aestivum*) specific probe was added to the hybridization mix. The plasmid containing the 2.4 kb fragment of 18S rRNA gene from *Cucurbita pepo* [36] was labelled with Cy3 using the Nick Translation Mix (Roche Applied Science) according to manufacturer’s protocol. The plasmid pTa794, containing a 410 bp *Bam*HI fragment of 5S rRNA gene and spacer region of wheat [37] was labelled with Fluoro-Red-dUTP (Amersham) by PCR and used as a 5S rDNA probe. A universal primer P26S (5′-GATCCACTGAGATTCAGCCC-3′) and a primer specific for wheat IGS -TIGS (5′-TGAATCCACTTGCCTCAAATAGT-3′) - were used to amplify a 508 bp fragment of wheat intergenic spacer (IGS) and label it with digoxigenin. The PCR mixture consisted of 1×PCR buffer, 2 mM MgCl_2_, 0.2 mM dNTP, 1 mM digoxigenin-dUTP, 0.2 mM of each primer and 1U of Taq Polymerase (Promega). The cycling conditions were as follows: 95°C for 5 min and 35 cycles of 30 s at 94°C, 30 s at 55°C, 1.5 min at 72°C with a final elongation step of 5 min at 72°C. The oak and triticale chromosomes together with the probes were denatured simultaneously for 5 min at 80°C or 2.5 min at 70°C, respectively, and were then left to hybridize overnight in a humid chamber at 37°C. After post-hybridization stringent washes, the slides were counterstained with 0.5 µg/mL DAPI for 8 min and mounted in antifade (DAKO Fluorescent Mounting Medium). In case of digoxigenin labelled rDNA probe, the slides were washed in 2×SSC, blocked for 30 min in 5% BSA (w/v) in 4×SSC/0.01% Tween (v/v) and incubated in 1∶125 dilution of FITC-conjugated anti-digoxigenin antibody (Roche), washed in 4×SSC/0.01% Tween (v/v), counterstained and mounted as previously described.

### Immunofluorescence

For immunodetection of 5-mC, the slides were denatured in 70% formamide in 2×SSC at 70°C for 2 min and dehydrated through an ice-cold ethanol series. The IF of 5-mC was done as described previously [38]. Briefly, after blocking, the slides were incubated in a 1∶200 dilution of anti-5mC antibody (Abcam, ab 10805) for 1 h at 37°C, washed and incubated in a FITC-conjugated secondary antibody (Abcam, ab 6785) for 1 h at 37°C. Finally, the slides were washed, counterstained with DAPI and mounted in ProLong Gold Antifade (Invitrogen). In combined immunofluorescence and FISH (IFF) experiments, following incubation with the secondary antibody to detect 5mC, the slides were dehydrated through an ethanol series and treated with RNase A solution (50 µg/mL in 2×SSC) for 1 h at 37°C. After washing in 1×PBS, the slides were post-fixed in 4% (w/v) paraformaldehyde for 30 min at room temperature, washed in 1×PBS, dehydrated through an ethanol series, dried for 30 min at 60°C and rehydrated for 10 min in 1×PBS prior to FISH detection. For immunodetection of histone marks, a protocol of Vičić *et al.* [38] was followed. Briefly, after blocking, the slides were incubated with primary antibodies prepared in the following dilutions: 1∶50 anti-H3K9ac (Abcam, ab 10812), 1∶100 anti-H3K27me2 (Abcam, ab 24684), 1∶200 anti- H3K9me1 (Abcam, ab 89906) and 1∶200 anti-H3K4me3 (Abcam, ab 71998) for 1 h at 37°C. The slides were washed and incubated for 1 h at 37°C with 1∶200 dilution of either Cy3- or FITC-conjugated secondary antibody (Abcam, ab 6939 and ab 6785). Finally, the slides were washed, counterstained with DAPI and mounted in antifade (DAKO Fluorescent Mounting Medium). For the combination of histone immunodetection and FISH, IF was carried out first and the slides were post-fixed as described previously. The hybridization mixture consisted of 50% (v/v) formamide, 10% (w/v) dextran sulphate, 50 mM NaH_2_PO_4_ and 1.5–2.0 ng/µL Cy3 labelled 45SrDNA probe in 2×SSC. The chromosomes and the probe were denatured simultaneously at 80°C for 5 min and were left to hybridize overnight in a humid chamber at 37°C. After post-hybridization stringent washes, the slides were counterstained with 0.5 µg/mL DAPI for 8 min and mounted in antifade (DAKO Fluorescent Mounting Medium).

### *In situ* hybridization on ultra-thin sections

Two different probes were used for ISH, both containing ribosomal genes of *Cucurbita pepo*. pRZ18 is a clone of the 2.4 kb *Hind*III fragment containing the whole 18S rRNA gene together with the ITS1 and 400 bp of the intergenic spacer. pRZ25 is a clone of the 2.9 kb *Hind*III fragment containing the 5.8S rRNA gene and ITS2, while the 25S rRNA gene lacks 200 bp of the 3′ end [36]. The probes were labelled with biotin (Boehringer Mannheim) by nick translation. The probe solution was prepared by mixing either 18S or 25S rDNA in 50% formamide, 10% dextran-sulphate, 0.1% SDS, 250 µg/mL salmon sperm and 2×SSC to a final probe concentration of 2–4 ng/µL.

Prior to hybridization, grids were treated with 100 µg/mL of RNase for 1 h at 37°C and with 5 µg/mL proteinase K (Sigma) for 10 min at 37°C, washed in 2×SSC supplemented with 50 mM MgCl_2_ and then re-fixed in freshly depolymerized 4% (w/v) paraformaldehyde for 5 min, followed by rinsing in 2×SSC. For hybridization, grids with ultra-thin sections were put into a drop of the denatured probe (10 min incubation at 70°C), covered with a coverslip and then put again at 72°C for 10 min to denature DNA on ultra-thin sections. Grids were then transferred into a humid chamber at 37°C to allow hybridization overnight. Post-hybridization stringent washes were performed as follows: 1×5 min in 2×SSC, 1×10 min in 50% formamide, and 2×5 min in 2×SSC at 40°C and then 2×5 min in 2×SSC and 1×5 min in PBS/Tween at room temperature. Grids were then treated with a BSA blocking solution for 5 min at room temperature and incubated in streptavidin/horseradish peroxidase (HRPO, DAKO) in PBS/Tween buffer (1∶500) for 1h at 37°C. After incubation, the grids were rinsed in PBS/Tween 3×5 min. The signal was visualized by diaminobenzidine (DAB) reduction; grids were incubated for 20 min at 4°C in 50 mM Tris-HCl, pH 7.4, 0.05% (w/v) DAB and 0.015% (v/v) fresh hydrogen peroxide. Reaction was stopped by rinsing with water several times and air-drying. Ultra-thin sections on grids were contrasted with a 4% (w/v) aqueous solution of uranyl-acetate and examined at 60 kV using a Zeiss EM 10A electron microscope.

### Evaluation of rRNA transcription level

To determine the expression levels of 18S rRNA genes and Actin 3 following treatment with 5-aza-2′-dC, RNA from the oak root tips was extracted according to Manickavelu [39] and purified by DNase I (Sigma). Reverse transcription reactions were performed using SuperScript III Reverse Transcriptase (Invitrogen) according to the manufacturer’s protocol, using the specific primer pair for reverse transcription of 18S rRNA (ITS1cr- GTTCTGCTGTGCAGGTTTCG; PIGS2cr- CTACCTGGTTGATCCTGCCA) and the Oligo(dT)12–18 Primer (Invitrogen) for Actin 3. The obtained cDNA was further amplified by PCR using the same primer pair for amplification of 18S rRNA and Act3F (GATTTGGCATCACACTTTCTACAATG) and Act3R (GTTCCACCACTGAGCACAATG) for Actin 3 amplification. The cycling conditions were as follows: 94°C for 2 min, 35 cycles of 30 s at 95°C, 1 min at 55°C and 2 min at 72°C for amplification of 18S cDNA; and 94°C for 3 min, 35 cycles of 45 s at 94°C, 45 s at 58°C and 1.5 min at 72°C for amplification of Actin 3. After electrophoresis of PCR products in 1% agarose gel and staining with ethidium bromide, gel image was captured using the Kodak EDAS 290 system with an Uvitec UV transilluminator. Bands were quantitated using the ImageJ software. Background was removed by the “rolling ball” method, which was followed by densitometry using the gel analysis module. All bands (18S rRNA and Actin3) were from the same gel, so the comparison was done by estimating uncalibrated integrated optical density expressed in arbitrary units. Direct analysis of the bands using the “region of interest” measurement was conducted to double-check and confirm the results of the gel analysis module.

### Image analysis and statistical analysis

For microscopy analysis, FISH images were captured using an epi-fluorescence microscope Axiovert 200 M (Zeiss, Germany), while IF and IFF images were captured using a confocal microscope Leica Microsystems TCS SP2 AOBS (Heidelberg, Germany), both coupled with CCD cameras. Optical sections were acquired by confocal microscope using integrated software. Images were processed and merged in ImageJ. Prior to co-localization analyses, images were deconvolved using 3D Parallel Iterative Deconvolution plug-in for ImageJ. Co-localization analyses were performed on 6-stack images by measuring the Pearson’s coefficient (PC) using an ImageJ-based plug-in JACoP [40]. Integrated fluorescence intensity analysis was performed using an ImageJ-based plug-in that added together all voxel intensity values for a selected nucleus and the obtained values were normalized to the volume of the nuclei determined by DAPI staining. Parameters for rDNA loci localization as well as nuclear and nucleolar volumes were obtained using ImageJ tools. Distance from nucleolus and periphery was normalized to nuclear radius. GraphPad Prism 5.0 software was used for statistical analyses. All analyses were carried out using the Mann-Whitney non-parametric t-test.

Confocal Z-stacks from the co-localization study were used for size comparison between the major (NOR-1) and the minor (NOR-2) rDNA locus. In total, 81 loci were analyzed (37 NOR-1 and 44 NOR-2). Images were analyzed using the ImageJ software [41]. Integrated density of each feature was measured across the Z-stack, with one measurement per optical section. No normalization was required, since all images had intensities running in the full 8-bit range (0 to 255), which was confirmed by the fact that no apparent variation in the overall intensity could be detected between images. At each section, the integrated optical density (OD) was multiplied by the area represented by one pixel to arrive at intensity in arbitrary units normalized to magnification. The section with the largest OD value was identified and taken as the cross-sectional area of the feature. Integrated OD by volume for each feature was obtained by adding OD of all the corresponding optical cross-sections, and multiplying the obtained value by the voxel volume. In the same manner, we quantitated the integrated volume of the condensed and decondensed NOR-1 in the six nuclei where these features were completely represented and clearly visible. The percentage of the decondensed fraction was calculated as the ratio between the integrated OD of the decondensed chromatin to the sum of the integrated OD of the decondensed and the integrated OD of the condensed fraction of the same NOR-1 site.

NOR size data were processed using the R statistical software [42]. Central 95% of the size range for NOR-1 and NOR-2 (between the quantiles 2.5% and 97.5%) were determined using the nonparametric Harrell-Davis quantile estimator [43] implemented in the R package “Hmisc” [44]. The 90% confidence intervals for the quantiles were estimated by bootstrapping [45] implemented in the R package “boot” [46].

## Results

### NOR-1 and the NOR-2 can be separated by their size

**Figure 1:**
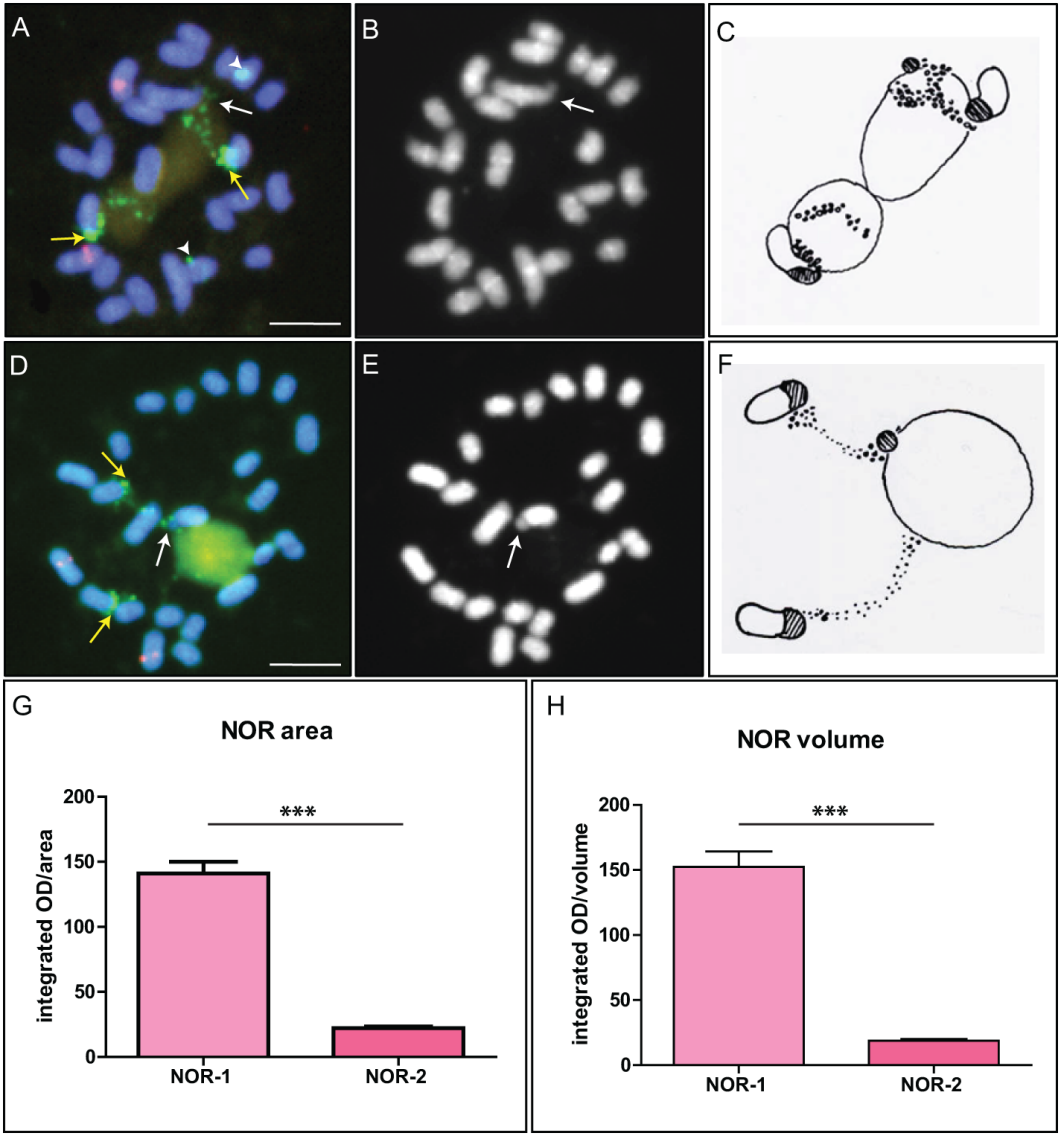
Fluorescence *in situ* hybridization (FISH) on metaphase chromosomes of *Q. robur* using 18S and 5S rDNA probes. FITC green signals represent the 18S rDNA probe, and Fluoro-Red signals correspond to the 5S rDNA probe (not relevant in this study). A. NOR-1 locus (arrows) is located terminally on a submetacentric chromosome pair. NOR-2 (arrowheads) resides near centromere of a small metacentric chromosome. Observe the signal size and strength dimorphism of NOR-2 sites. The rDNA knob (localized FISH signal) is situated proximally on one site (A and D, yellow arrow), and the rest of rDNA chromatin is decondensed (punctuate FISH signal). On another site, rDNA knob is present both at the proximal and the terminal (satellite) part of the site (white arrows, A and D) and the dispersed rDNA fraction resides between the two (visible as a punctuate FISH signal corresponding to SC). B and E. Satellites (distal part of the NOR-1 site) are indicated with arrows in DAPI stained metaphases. C and F. Schematic representations of chromatin topology at the major rDNA locus corresponding to figures A and D. G. and H. Graphical representations of measured NOR-1 and NOR-2 sizes (G, arbitrary OD units normalized to pixel area) and volume (H, arbitrary OD units normalized to voxel volume) demonstrate that the two loci can be unambiguously identified. Number of asterisks indicates p<0.0001; error bars represent the standard error of the mean. Scale bar is 5 µm.

Four FISH signals representing two 45S rDNA loci, referred to as the major (NOR-1) and the minor (NOR-2) locus, were identified in interphase nuclei of the common oak root tip cycling cells, consistent with our previous findings [35]. The NOR-1 locus is positioned terminally on the short arm of a subtelocentric chromosome pair, while the NOR-2 locus resides at a paracentromeric region of a medium-sized metacentric chromosome pair (Fig. 1A and D). Even though molecular distinction of the two loci is still not possible because of the high similarity of rRNA copies within both sites [47] and our unpublished data), the difference in size alone was sufficient for discrimination between NOR-1 and NOR-2 locus. Ranges for both cross-section and integrated volume were completely free of overlap between NOR-1 and NOR-2 making their identification unambiguous. Modeling the spread of NOR-1 and NOR-2 cross-section and volume data by calculating quantiles in a manner similar to calculation of reference intervals in clinical chemistry [48] demonstrated that it was highly unlikely to confuse the two sites.

The mean integrated OD by area (in arbitrary units, normalized by pixel area) of the largest cross-section of NOR-1 was 139.34, while it was 22.11 for NOR-2 and the difference in measured values was statistically significant (p<0.0001) (Fig. 1G). The observed OD by area range was 65.29 to 286.01 for NOR-1 and 7.95 to 41.67 for NOR-2. The central 95% (from 2.5% to 97.5%) of the OD by area range for NOR-1 was 68.76 (90% confidence interval: 65.37 to 76.05) to 263.35 (90% confidence interval: 218.19 to 285.20), while for NOR-2 it was 8.79 (90% confidence interval: 7.99 to 11.98) to 39.93 (90% confidence interval: 36.55 to 41.62).

The mean integrated OD by volume (in arbitrary units, normalized by voxel volume) of NOR-1 was 150.91, while it was 18.59 for NOR-2 (Fig. 1H), the difference between the two being statistically significant (p<0.0001). The observed OD by volume range was 62.04 to 301.35 for NOR-1 and 4.53 to 39.42 for NOR-2. The central 95% (from 2.5% to 97.5%) of the OD by volume range for NOR-1 was 63.15 (90% confidence interval: 62.07 to 66.16) to 299.65 (90% confidence interval: 293.26 to 301.31), while for NOR-2 it was 5.66 (90% confidence interval: 4.57 to 7.58) to 38.10 (90% confidence interval: 32.65 to 39.37). Neither the observed range, nor the 90% confidence limits of the central 95% of the observed range (quantiles 2.5% to 97.5%) were overlapping, demonstrating good separation of NOR-1 and NOR-2 by size alone. Exact measurements are given in the supplementary Table S1 and examples of Z-stacks can be found in Fig. S2.

### rDNA chromatin topology

While rDNA chromatin of the minor locus was always highly condensed (Fig. 1A, arrowheads), NOR-1 locus showed two types of rDNA chromatin organization – a highly condensed rDNA chromatin (rDNA knob, Fig. 1A and D, arrows; C and F, dashed area on chromosome drawings) and a more decondensed rDNA chromatin (Fig. 1A and D, FITC-green dispersed signal; C and F, dots on schematic presentation). This dispersed FISH signal was not homogenous but comprised smaller and larger dots interconnected with thin threads (Fig. 1A and D). The dispersed FISH signal was associated with the nucleolus/nucleoli (Fig. 1 A, C, D and F) and probably contains transcriptionally active rRNA genes, so that rDNA chromatin stays less condensed even during (pro)metaphase.

Two homologous NOR-1 sites showed dimorphism regarding chromatin topology and the type of chromatin organization was not fully random as metaphase NOR-1 sites showed the same dimorphism in majority of the cells within an individual (Table 1). Even though both sites contained both rDNA knob and dispersed rDNA fraction, their arrangement was different. At one site, the rDNA knob was present at the proximal end of the site (Fig. 1A and D, yellow arrows) and the rest of rDNA chromatin was dispersed (see also Fig. 1C and F), while on the other site the condensed chromatin resided on both the proximal and the distal end (i.e. satellite) of the locus and the dispersed rDNA fraction was spread between the two (Fig. 1A and D, white arrows, see also C and F).

**Table 1:** Different patterns of rDNA chromatin organization at NOR-1 sites.

Individual	A	B	F	T
**1**	75	3	0	78
**2**	80	0	24	104
**3**	101	2	5	108
**4**	109	1	3	113
**5**	83	1	2	86
**6**	136	0	3	139
**7**	102	0	3	105
**8**	106	0	7	113
**9**	78	0	2	80
**10**	50	2	11	63
**11**	66	4	11	81
**12**	54	0	6	60

A number of cells with a particular rDNA chromatin topology at the NOR-1 sites was observed. A - one NOR-1 site shows a terminal rDNA knob and a proximal dispersed rDNA fraction, corresponding to decondensed rDNA chromatin, while another NOR-1 site contains a rDNA knob at the proximal and the distal (satellite) end with dispersed rDNA chromatin spreading between the two; B - one NOR-1 site shows proximal rDNA knob and a terminal dispersed rDNA fraction, the other site shows only condensed rDNA chromatin at the satellite, while rDNA chromatin from the proximal part of the site is dispersed; F - fusion of the sites, i.e. very close positioning of proximal rDNA knobs in relation of each other, T - total number of analyzed cells.

We tried to determine relative abundance of each type of rDNA chromatin fraction - condensed (rDNA knob) and decondensed (dispersed FISH signal) - on confocal Z-stacks as described in the Materials and methods section. Of the six nuclei where the decondensation fraction could be measured, two nuclei showed one fully condensed NOR-1 site, while the second site was 75.3% and 52.7% decondensed. The sum of the decondensed and the condensed fraction closely matched the fully condensed NOR-1 site (within 25%). The other four measured nuclei had a single visible NOR-1 site, which was partially decondensed. The decondensed fraction was 61.9%, 45.3%, 62.7% and 57.7%. The measured decondensation corresponded to approximately half of an NOR-1 site. In cases when there was only a single NOR-1 site visible, it was unclear whether the decondensed material was a part of the visible NOR-1 site, or represented the other which could not be seen due to complete decondensation. Examples of Z-stacks showing partial decondensation are shown in the supplementary Figure S3.

### Subnuclear distribution of rDNA chromatin

**Figure 2:**
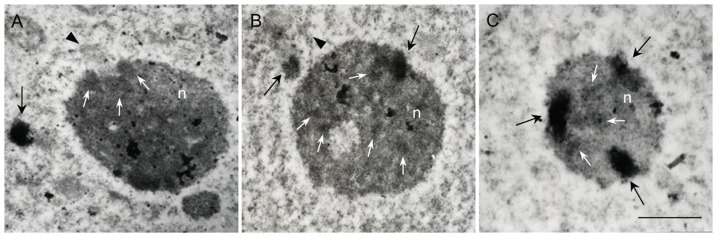
Electron micrographs of *in situ* hybridization with biotin labeled 18S rDNA probe on sections through root tip meristematic tissue of *Q. robur*. Hybridization area was detected with DAB. Labeled condensed chromatin lies outside or close to the nucleolus (A and B, black arrows), protruding into the nucleolus (B, black arrow) and inside the nucleolus near the nucleolar border (C, black arrows). Diffuse hybridization signal that corresponds to completely decondensed DNA is seen inside the nucleolus (A, B, C, white arrows). Unlabeled chromatin corresponding to the NOR-bearing chromosome can be seen near the nucleolus (B, black arrowhead) and protruding into it (A, black arrowhead). Letter «n» marks the nucleolus. Scale bar is 2.5 µm.

The sites of 18S rDNA probe hybridization were visualized on ultra-thin sections by electron-opaque deposits of reduced DAB. ISH signal corresponding to condensed rDNA chromatin (rDNA knob) was seen outside of the nucleolus (Fig. 2A, black arrow) and on the nucleoplasm-nucleolus boundary i.e. protruding into the nucleolus (Fig. 2B and C, black arrows). Beside the localized signal, diffuse signals were visible inside the nucleolus (Fig. 2A, B and C, white arrows) corresponding to the decondensed rDNA chromatin fraction. In some other sections, unlabeled chromatin protruded into the nucleolus (Fig. 2A, black arrowhead) and near that site, a diffuse signal could be seen (Fig. 2A, white arrows). Unlabeled chromatin was also noticed adjacent to nucleolus (Fig. 2B, black arrowhead), probably corresponding to the chromatin of a NOR-bearing chromosome.

Mostly a single nucleolus was seen in EM sections, perhaps because of the random sectioning of the tissue, while two nucleoli were seen only rarely. A smaller body of the same consistence, probably representing the nucleolar-associated body, appeared often near the nucleolus (Fig. 2A).

### Positioning of NOR-1 anad NOR-2 loci in the interphase nucleus of the common oak and triticale

**Figure 3:**
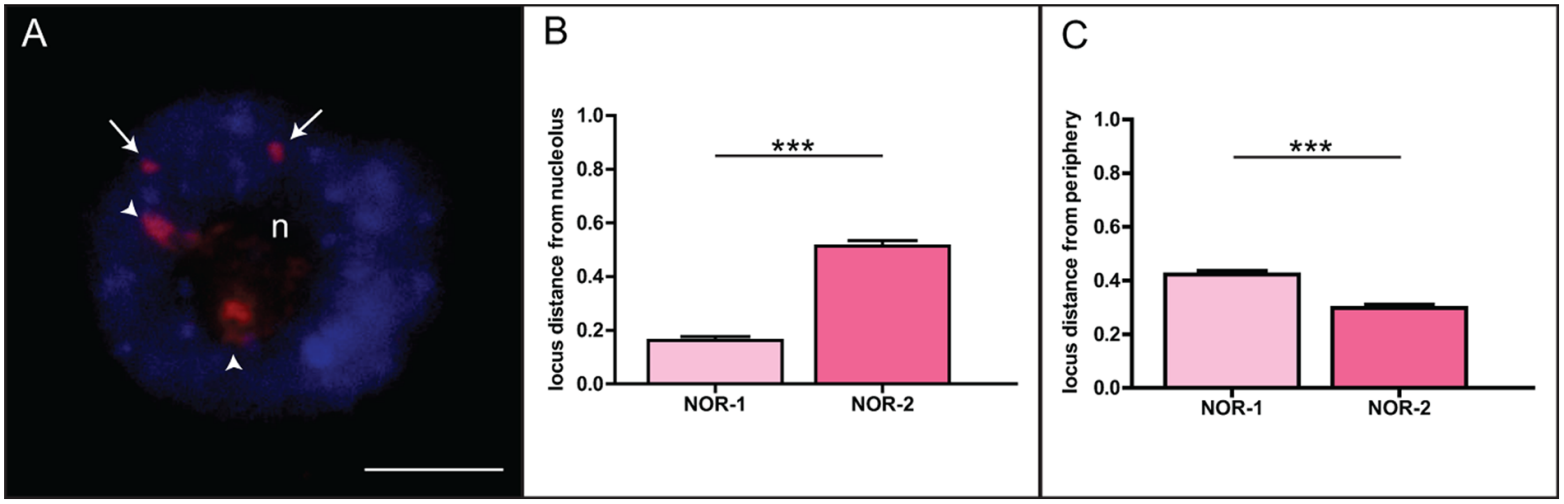
Position of NOR-1 and NOR-2 in interphase nucleus of *Q.robur*. A. Major 45S rDNA sites (NOR-1, arrowheads) are associated with the nucleolus while minor 45S rDNA sites (NOR-2, arrows) are positioned at the nuclear periphery. Statistical analysis of measured distances from the nucleolus (B, N = 220) or the nuclear periphery (C, N = 240) reveals differences between the two rDNA loci. Number of asterisks indicates p<0.0001; error bars represent the standard error of the mean. Letter «n» marks the nucleoli. Scale bar is 5 µm.

We aimed to measure the distance between NOR-1/NOR-2 sites and the nucleolus/nuclear periphery after FISH with the 18S rDNA probe. After counter-staining of interphase chromatin with DAPI, the nucleolus appeared as a dark unstained region. The major 45S rDNA (NOR-1) sites (Fig. 3A, arrowheads) were almost always associated with the nucleolus whereas the minor 45S rDNA (NOR-2) sites (Fig. 3A, arrows) were found predominantly located at the nuclear periphery. This observation was further confirmed by statistical analysis of the measured distances from the nucleolus and the nuclear periphery (Fig. 3B and C). Both NOR-1 sites were found in close proximity to the nucleolus, whereas the minor sites were significantly further away from it and, correspondingly, closer to the nuclear periphery (Fig. 3C). Nucleoli constituted up to 18% of the nuclear volume and were placed centrally, on average one quarter of nuclear diameter away from the nuclear center.

**Figure 4:**
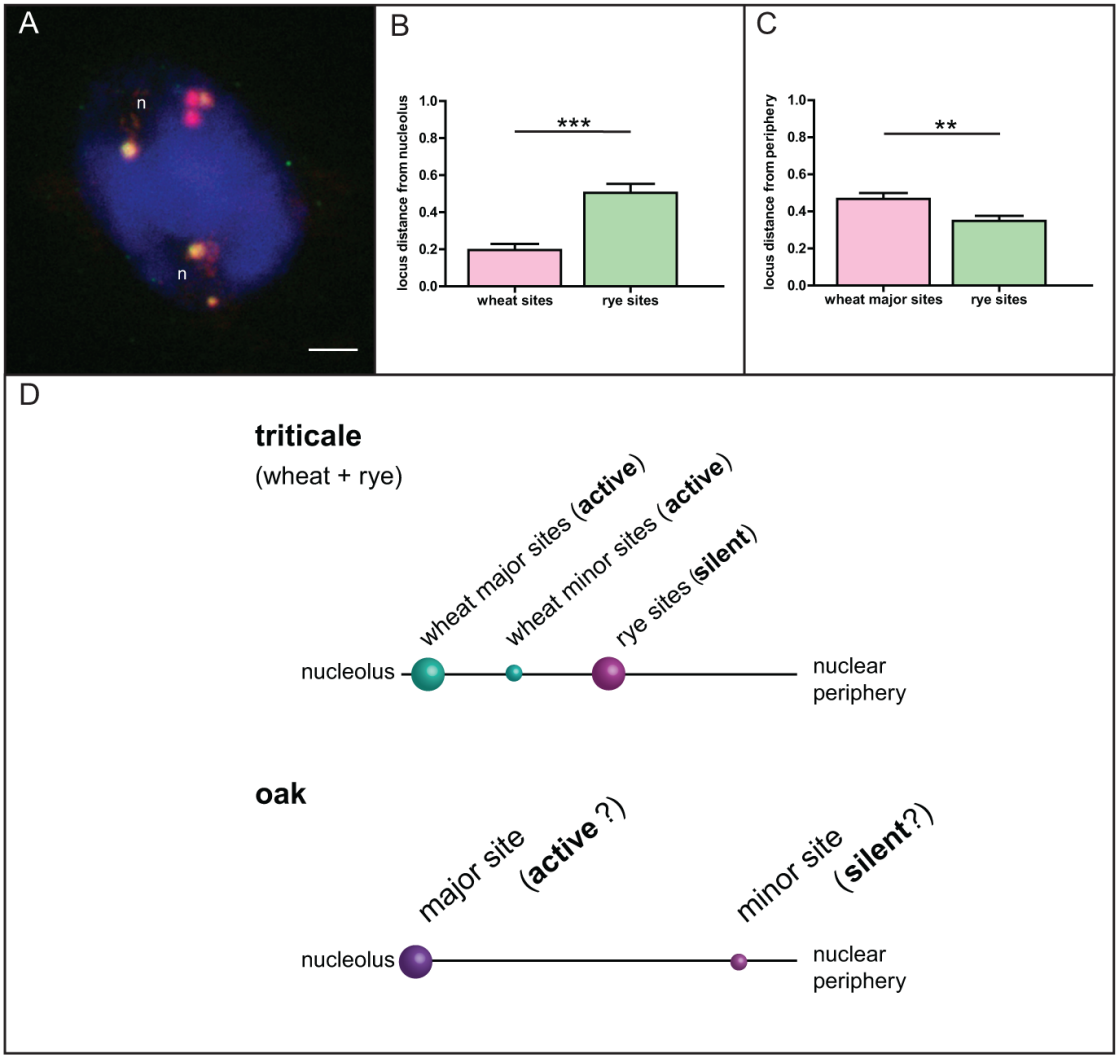
Correlation between the position of a NOR locus relative to the nucleolus and its transcriptional activity. A. Locations of wheat (yellow-green) and rye (red) NOR loci in nuclei of triticale revealed by FISH. Statistical analysis of measured distances from the nucleolus (B, N = 42) or the nuclear periphery (C, N = 42) revealed significant differences between wheat and rye rDNA loci, concordant with the established transcriptional activity of the wheat loci and silencing of the rye locus due to nucleolar dominance. Number of asterisks indicates p<0.01 (**) and p<0.0001 (***); error bars represent the standard error of the mean. D. The graphical illustration of the correlation between the position of a NOR locus in relation to the nucleolus and its transcriptional activity in triticale and the common oak. Letter “n” marks the nucleoli. Scale bar is 5 µm.

To test whether the proximity of an rDNA locus to the nucleolus is indicative of its transcriptional activity, we performed an additional analysis in triticale, a hybrid between rye (*Secale cereale*) and wheat (*Triticum aestivum*), with clearly differentiated NOR activities. The expression of 45S rDNA locus originating from rye is suppressed, while the two wheat-derived 45S rDNA loci are expressed [9]. Rye- and wheat-derived sites were detected using a combination of FISH probes that allowed distinguishing the two (Fig. 4A). We found that transcriptionally silent rye-derived 45S rDNA sites were significantly further away from the nucleolus and correspondingly closer to the periphery when compared to the wheat counterparts (Fig. 4A and B). Based on these results we designed a graphical model depicting relative positions of each of the rDNA loci in nuclei of both species (Fig. 4D). If an analogy to the triticale hybrid system is considered, where there is a strong correlation between rRNA gene transcriptional activity and the locus-nucleolus association, then our model suggests that in oak only the major 45S rDNA sites are transcriptionally active.

### Changes in NOR-1 and NOR-2 methylation status and nuclear positioning following treatment with 5-aza-2′-dC

**Figure 5:**
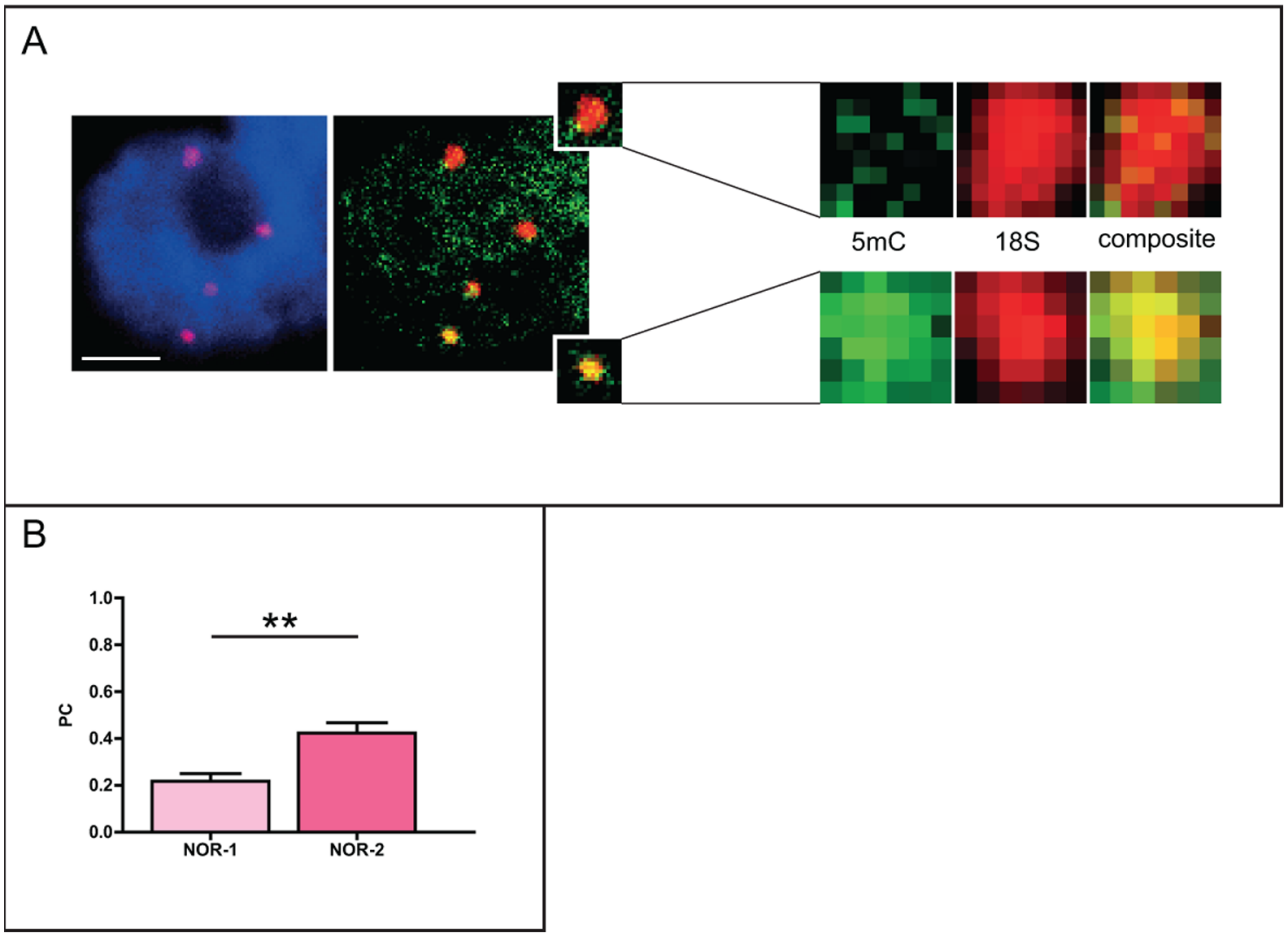
Level of DNA methylation in NOR-1 and NOR-2 of *Q.robur*. A. Immunofluorescence using an antibody against 5-mC coupled with FISH using the 18S rDNA probe revealed the presence of 5-mC in both 45S rDNA loci. B. Co-localization analysis of 5-mC and FISH signals showed stronger co-localization in minor rDNA sites (NOR-2) when compared to major rDNA sites (NOR-1); N = 26. Number of asterisks indicates p<0.01; error bars represent the standard error of the mean. Letter “n” marks the nucleoli. Scale bar is 5 µm.

To gain further insight into the possible differential activity of the minor and the major rDNA loci in *Q. robur*, we performed a co-localization analysis following combined immunodetection of 5-mC and FISH with the 18S rDNA probe. Visual inspection revealed abundance of 5-mC within the minor NOR-2 sites, while NOR-1 sites were largely devoid of this modification (Fig. 5A). Co-localization analysis performed by estimating the Pearson’s coefficient further confirmed a high co-localization level between 5-mC and the minor 45S rDNA sites (Fig. 5B).

**Figure 6:**
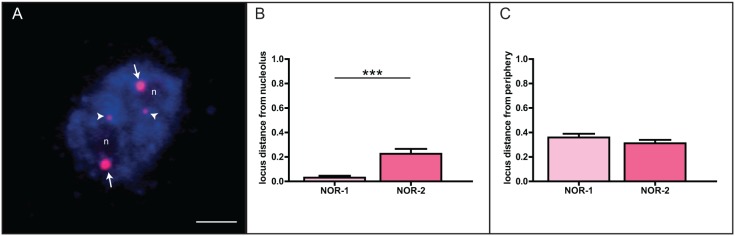
Nuclear repositioning of NOR-2 of *Q.robur* following inhibition of DNA methylation. A. After the treatment with 5-aza-2′-dC, NOR-2 sites (arrowheads) repositioned much closer to nucleoli, as confirmed by statistical analysis (B). Statistical analysis shows equal distance of the both NOR loci from the nuclear periphery (C). Number of asterisks indicates p<0.0001; error bars represent the standard error of the mean. Letter “n” marks the nucleoli. Scale bar is 5 µm.

To ascertain whether the disruption of DNA methylation has an effect on the spatial organization of the oak 45S rDNA loci, we treated root tips with the DNA methyltransferase inhibitor 5-aza-2′-dC. The treatment resulted in a significant decrease in global 5-mC levels (Fig. S1B), which we further confirmed by integrated fluorescence intensity analyses (Fig. S1C). Additionally, the reduction of global DNA methylation levels coincided with reorganization of nuclear architecture. After the treatment, chromatin seemed less dense and the nucleolus occupied a larger volume of the nucleus–22% in treated *versus* 18% in untreated nuclei – or two nucleoli appeared instead of one (in 44% of nuclei analyzed). Interestingly, we could observe a clear re-localization of the NOR-2 sites towards the nuclear center and the nucleoli, however it appeared that they never associated with the nucleolus/nucleoli (Fig. 6A, arrowheads, and B). If the nuclei had two nucleoli, each of the NOR-1 sites was associated with an individual nucleolus (Fig. 6A, arrows) indicating preserved dominant contribution of NOR-1 sites to the nucleolus formation.

**Figure 7:**
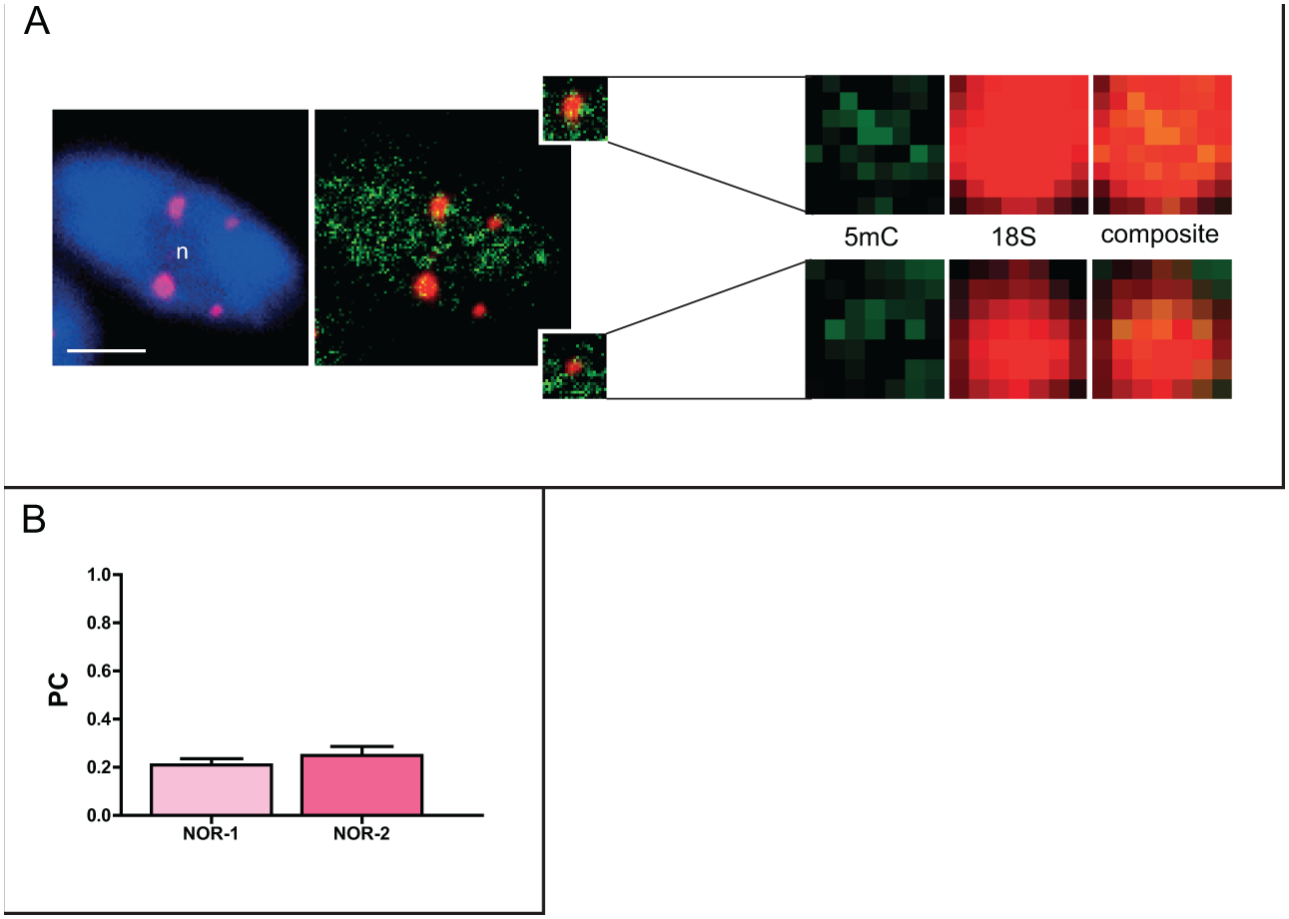
Decrease in DNA methylation level in NOR-1 and NOR-2 of *Q.robur* following treatment with 5-aza-2′-dC. A. Immunofluorescent detection of 5-mC coupled with FISH using 18S rDNA probe revealed the reduction of 5-mC levels in NOR-2 sites. B. Similar levels of 5-mC in both NOR loci were confirmed by co-localization analysis. Error bars represent the standard error of the mean. Letter “n” marks the nucleoli. Scale bar is 5 µm.

The epigenetic treatment significantly decreased the DNA methylation level within the NOR-2 sites (Fig. 7A). Interestingly, when comparing the co-localization level of FISH signals of the major or the minor rDNA sites with 5-mC, we could not observe any difference between the loci (Fig. 7B). It was evident that the treatment did not affect the already low methylation levels of the major 45S rDNA sites (PC = 0.2; Figs. 5B and 7B), but rather induced a decrease in the 5-mC level within the minor 45S rDNA locus (from PC = 0.42 to PC = 0.23 before and after the treatment, respectively).

### Change in NOR-1 and NOR-2 histone marks following inhibition of DNA methylation

**Figure 8:**
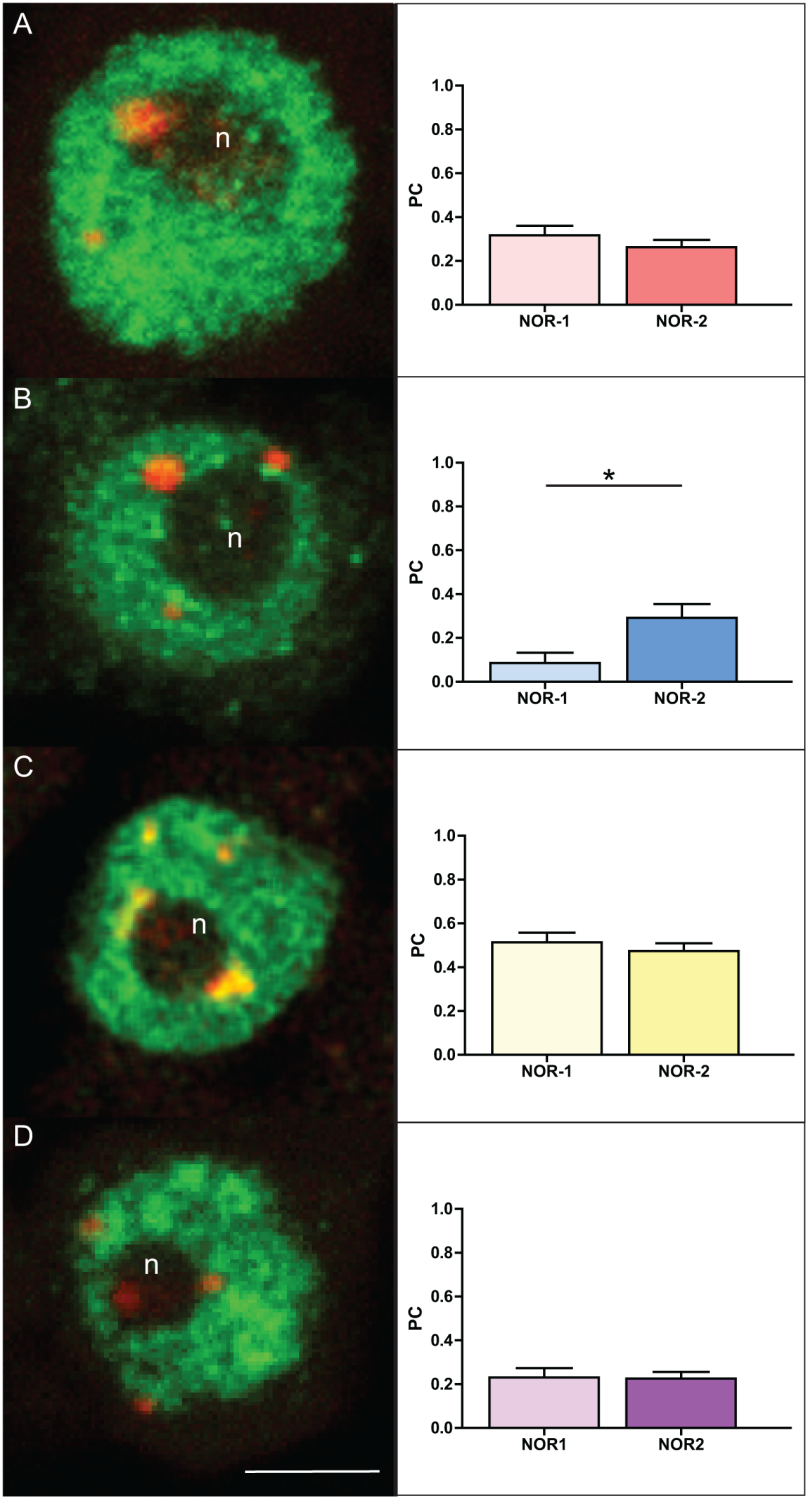
Histone modifications associated with NOR-1 and NOR-2 loci in root tip cycling cells of *Q.robur*. IFF detection of H3K9ac (A), H3K4me3 (B), H3K9me1 (C) and H3K27me2 (D) and graphs showing Pearson’s coefficient of co-localization of respective histone marks with NOR loci. Green signals correspond to immunofluorescence of histone antibodies and red signals represent 18S rDNA FISH signals. Number of asterisks indicates p<0.05; error bars represent the standard the error of the mean. Letter “n” marks the nucleoli. Scale bar is 5 µm.

We performed multiple IFF experiments using antibodies against histone marks shown in *Arabidopsis* to be characteristic for open (H3K9ac, H3K4me3) and closed (H3K9me1, H3K27me2) conformation of rDNA chromatin [8], in order to reveal histone signatures of rDNA chromatin in oak cycling cells. For all histone marks 20–30 NOR-1 and NOR-2 sites were analyzed. A moderate association of both rDNA loci was found with the H3K9ac (NOR-1 = 0.317; NOR-2 = 0.263, Fig. 8A) and H3K27me3 (NOR-1 = 0.230; NOR-2 = 0.225; Fig. 8D) histone marks. The strongest association of oak 45S rDNA loci was found for H3K9me1 (NOR-1 = 0.563, NOR-2 = 0.519; Fig. 8C). Interestingly, in case of the NOR-1 locus, this histone mark was confined to the rDNA chromatin knob, whereas the dispersed rDNA chromatin visible within the nucleolus was clearly devoid of the H3K9me1 signal. Surprisingly, H3K4me3 was the only histone mark differently represented in oak rDNA loci and it showed a stronger association with the NOR-2 locus (NOR-1 = 0.086; NOR-2 = 0.292; p = 0.0147; Fig. 8B).

**Figure 9:**
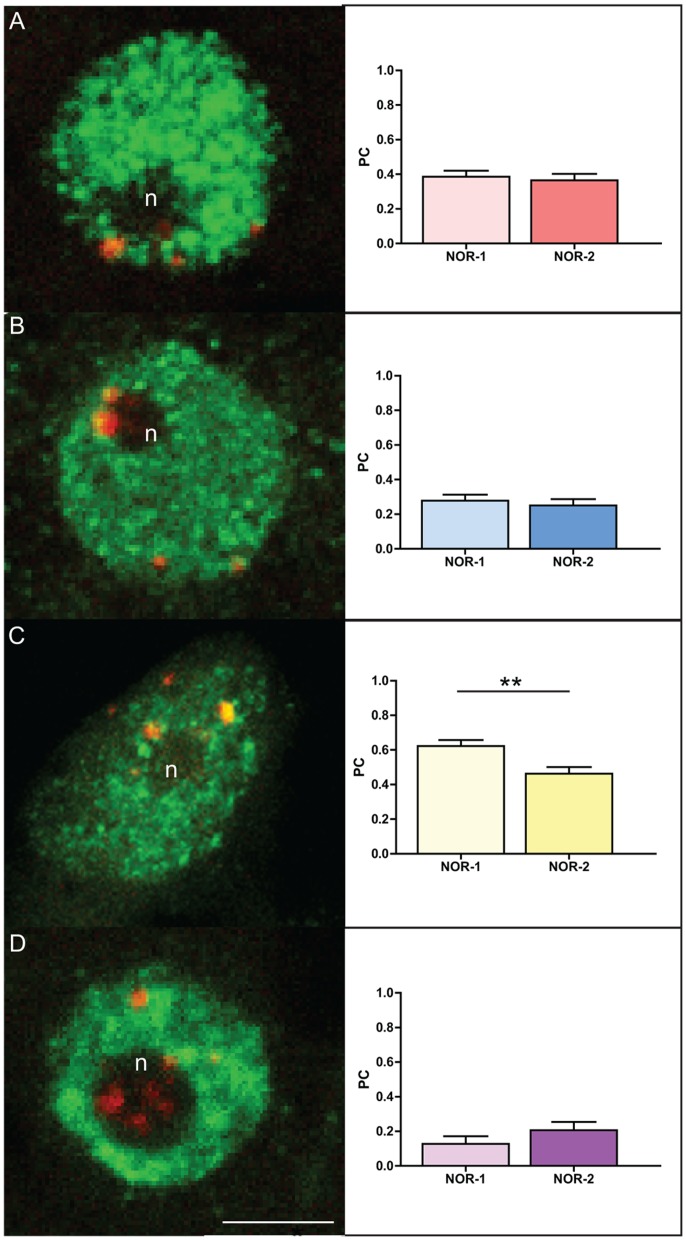
Histone modifications associated with NOR-1 and NOR-2 loci in root tip cycling cells of *Q. robur* after treatment 5-aza-2′-dC. IFF detection of H3K9ac (A), H3K4me3 (B), H3K9me1 (C) and H3K27me2 (D) and graphs showing Pearson’s coefficient of co-localization of respective histone marks with the NOR loci. Green signals correspond to immunofluorescence of histone antibodies and red signals represent 18S rDNA FISH signals. Number of asterisks indicates p<0.01; error bars represent the standard error of the mean. Letter “n” marks the nucleoli. Scale bar is 5 µm.

In order to see the effect of a change in global DNA methylation level on the distribution of the analyzed histone marks within the rDNA chromatin, we repeated the IFF analyses after treatment of oak seedlings with 5-aza-2′-dC (Fig. 9A – D). Again, 20–30 sites of NOR-1 and NOR-2 locus were analyzed for each histone mark. We found that two of the tested histone marks showed significant changes in their co-localization values. The treatment resulted in an increase of co-localization of H3K4me3 with the NOR-1 locus (control = 0.085; treatment = 0.278; p = 0.0041, Fig. 8B and 9B). It also caused a significant decrease of H3K9me1 co-localization in the NOR-2 locus compared to the NOR-1 locus (NOR-1 = 0.623; NOR-2 = 0.463; p = 0.0019, Fig. 9C) even though the individual PC values of NOR-1 and NOR-2 did not statistically differ from the control values. These results could indicate transcriptional reactivation of a previously inactive fraction of rRNA genes within both NOR loci. H3K9ac and H3K27me2 were not significantly altered following global DNA demethylation.

### Transcriptional reactivation of rRNA genes after treatment with 5-aza-2′-dC

**Figure 10:**
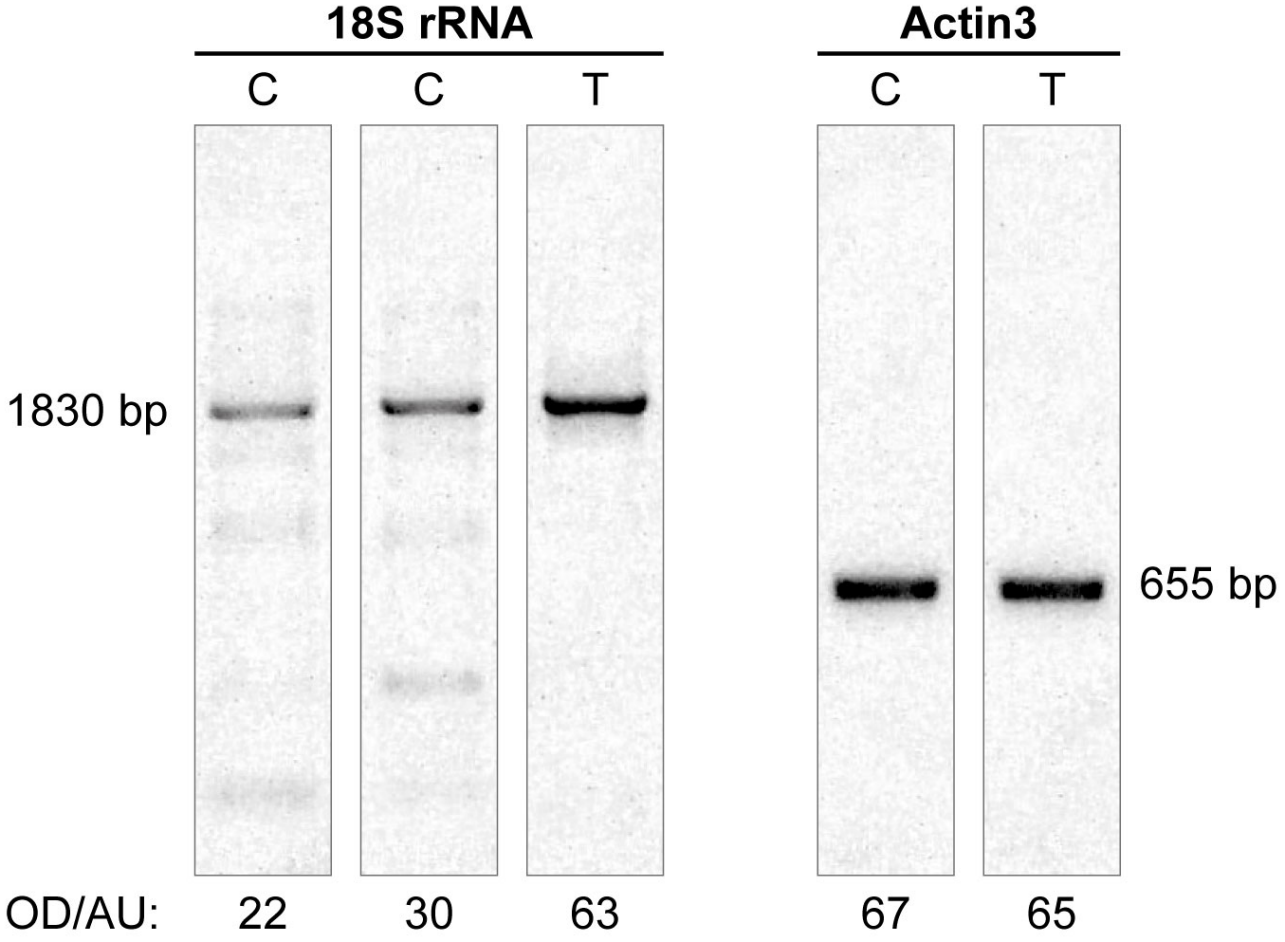
RT-PCR analysis of 18S rRNA transcription. RT-PCR analysis detected an increased level of 18S rRNA transcripts after treatment of root tips with 5-aza-2′-dC. C – control, T – treatment with 5-aza-2′-dC. OD/AU is optical density (in arbitrary units) of RT-PCR product peaks after electrophoresis in agarose gel and staining with ethidium bromide. The 18S rRNA (1830 bp band) transcription increased more than twofold after treatment with 5-aza-2′-dC, while the transcription level of the Actin3 gene (655 bp, used as control) remained essentially at the same level.

A global decrease in DNA methylation following 5-aza-2′-dC treatment resulted in rDNA loci redistribution and an increase in nucleoli volume/number, suggesting a potential increase in rRNA gene activity. To address this question, we performed a RT-PCR analysis. In 3 independent experiments we found more than a twofold increase in 18S rRNA transcription following the treatment, confirming that the observed epigenetic changes go hand in hand with the changes in transcriptional activity (Fig. 10). However, there is still no valid method to distinguish transcription arising from a particular rDNA locus in oak cycling cells.

### Silver staining

**Figure 11:**
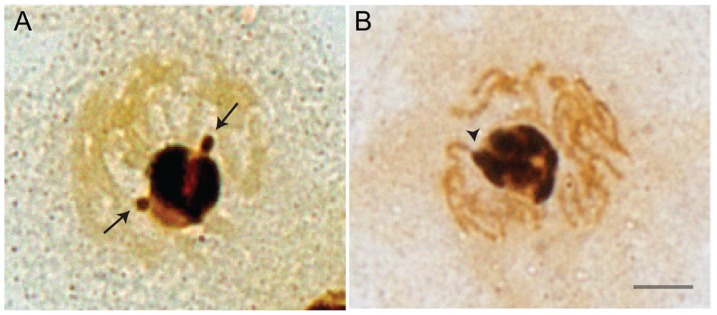
Silver staining of chromosomes and nucleolus in root tip cycling cells of *Q. robur.* Silver stained nuclei before (A) and after treatment with 5-aza-2′-dC (B) show strong silver deposition within the whole area corresponding to a single large nucleolus. A. rDNA knobs of both NOR-1 sites seen at the nucleolar periphery (arrows) show mild silver deposition. B. After treatment with 5-aza-2′-dC rDNA chromatin of the knob was decondensed, extending from the NOR-bearing chromosome to the nucleolus which was completely stained with silver (arrowhead). Scale bar is 5 µm.

Silver staining of oak chromosomes revealed a prominently stained nucleolus and two regions of silver precipitation with position and size corresponding to rDNA chromatin knobs of the NOR-1 sites. Therefore, silver staining enabled positive identification of NOR-1 sites positioned adjacent to the nucleolus (Fig. 11A arrows). Much stronger silver deposition was observed within the whole nucleolus, corresponding to the dispersed rDNA chromatin fraction of the NOR-1 locus containing actively transcribing rRNA genes (Fig. 11A). No other silver stained regions were visible on oak chromosomes suggesting that only the NOR-1 locus is involved in formation of the nucleolus and contributes in rRNA gene transcription. After treatment with 5-aza-2′-dC, no localized silver deposition corresponding to rDNA knob of the NOR-1 site was seen, suggesting decondensation of rDNA chromatin. Also, no additional silver stained sites were found, indicating that NOR-2 sites were not reactivated (Fig. 11B).

## Discussion

### NOR-1 and NOR-2 loci show different rDNA chromatin topology, distinct positions with respect to the nucleolus and different epigenetic signatures, suggesting their transcriptional status

The aim of this study was to deduce the transcriptional status of the major and the minor rDNA locus (NOR-1 and NOR-2) in root tip cycling cells of pedunculate oak by: 1) analyzing the association between a NOR and the nucleolus; (2) observing the rDNA chromatin topology; (3) staining NORs with silver; (4) comparing the presence/absence of DNA methylation and histone modifications at rDNA loci and (5) comparing levels of rRNA transcripts before and after the treatment with DNA methylation inhibitor 5-aza-2′-dC. Our data suggest that only rRNA genes residing within the major rDNA locus (NOR-1) are transcriptionally active under normal physiological conditions and the minor locus (NOR-2) probably stays transcriptionally silent. This situation resembles a phenomenon known as nucleolar dominance, where epigenetic differences determine and maintain the dominant and repressed sets of rRNA genes in hybrid species [3], [8], [18], [49]. The evidence supporting the silent status of the NOR-2 locus is several-fold:) (1) the NOR-2 sites never associated with the nucleolus, but instead were situated close to the nuclear periphery; (2) the rDNA chromatin of the NOR-2 locus always appeared highly condensed and no dispersed rDNA signal (corresponding to more decondensed rDNA chromatin fraction) was visible even following the epigenetic treatment; (3) NOR-2 locus showed no silver precipitation and (4) the NOR-2 sites were enriched for repressive (5-mC, H3K9me1) and deprived of permissive (H3K9ac and H3K4me3) epigenetic marks. By contrast, both sites of the NOR-1 locus were consistently associated with a large single nucleolus (rarely each site formed its own nucleolus), they consisted of dispersed rDNA chromatin fraction with peri- and intra-nucleolar position (seen both by light and electron microscopy), they were stained with silver and contained hypomethylated DNA.

### Localization of NORs, rDNA chromatin topology and transcriptional activity of 45S rRNA genes are in part regulated by cytosine methylation

Comparison of the triticale and *Q. robur* nuclei models, constructed on the basis of measured distances of NORs from the nucleolus/nuclear periphery, shows that thedistance from the nucleolus can be considered as an indication of NOR activity in pedunculate oak. In triticale, we have found that only NORs of wheat origin associate with the nucleolus, indicating a strong correlation between a NOR locus position and its activity. Therefore, positioning of the oak’s NOR-1 locus close to the nucleolus would suggest that rRNA genes within this locus are transcriptionally active, while NOR-2 sites that are closer to the nuclear periphery can be considered transcriptionally silent. Treatment with the methyltransferase inhibitor 5-aza-2′-dC causes global hypomethylation and reactivation of silent NORs in triticale, *A. thaliana* and *Brassica napus* [5], [7], [9], [50] indicating that DNA methylation is necessary for rRNA gene silencing. In oak root tip cycling cells, DNA hypomethylation caused by 5-aza-2′-dC treatment resulted in repositioning of NOR-2 sites closer to the nucleolus, an increase in nucleolar size and number and a twofold increase in 18S rRNA transcription. These results are in agreement with the results of Caperta and co-workers [19], who have demonstrated that 5-aza-2′-dC treatment causes reactivation of a silent NOR in rye, an increase in steady-state rRNA transcription accompanied with the enlargement of the nucleolus, decondensation of rDNA chromatin and relocation of NORs into the nucleolus.

rDNA chromatin within NOR-2 locus was highly methylated under normal physiological conditions of root tip cycling cells, and chromatin was marked with H3K9me and moderately with H3K27me2, both histone modifications characteristic for repressive rDNA chromatin. Treatment of the cells with 5-aza-2′-dC resulted in a decrease in cytosine methylation and H3K9me1 within NOR-2, both signs of a change from the repressive to the permissive chromatin state. Also, the treatment caused repositioning of NOR-2 locus much closer to the nucleolus. Nevertheless, we found no cytological evidence of NOR-2 transcriptional activity, i.e. no silver precipitation and/or rDNA decondensation. In fact, the presence/absence of an epigenetic modification itself in rDNA chromatin is not sufficient for determination of its transcriptional status, as it has been shown for *Arabidopsis*, a mutant for *AtHDA6* (histone deacetylase gene), that hyperacetylation is not concomitant with increased rRNA transcription [51].

Our results suggest that the increase in total transcription levels of rRNA genes in *Q. robur* after the treatment with 5-aza-2′-dC is rather due to up-regulation of the active fraction of genes in the NOR-1 locus or possibly reactivation of the silent portion of that locus, than reactivation of the NOR-2 locus. Muir and co-workers suggested that only a single rDNA family of the three divergent rDNA families identified in *Q. robur* is functional [52]. They found that the rate of sequence change was significantly elevated in the two rDNA gene families which are not expressed compared to the functional gene family and concluded that these two families exist as pseudogenes. Based on the combined results of expression analysis, relative rate tests and mutation spectrum analyses they suggested that nucleolar dominance is not suppressing the expression of two of the three rDNA clades present in *Q. robur* genome, but rather that these sequences have lost their function. The results from this study also suggest that NOR-2 associated rRNA genes have lost their function. In fact, Muir et al. [52] have proposed a scenario where three highly divergent rDNA families were brought together in the genome of *Q. robur* through an ancient hybridization process, which prevented concerted evolution to homogenize these sequences [53]. In a situation where there is little or no genetic exchange, nucleolar dominance could assure the expression from only a single rDNA family (i.e. NOR-1 locus in *Q. robur*). Since nucleolar dominance could be maintained over generations [10] there is a possibility that silenced rDNA family (i.e. rRNA genes from the NOR-2 locus in *Q. robur*) could have evolved as a pseudogene.

### Structural and functional domains within the major 45S rDNA locus (NOR-1)

Two different structural domains – a highly condensed (rDNA knob) and a dispersed heterogeneous rDNA chromatin fraction of peri- and intranucleolar position - co-exist within the major rDNA locus (NOR-1). The same has been shown for the active NOR in diploid rye [17], for the dominant *A. arenosa* NOR in the hybrid *A. suecica* [18] and for the dominant NOR of wheat origin in a wheat-rye hybrid [20]. These previous observations have suggested that the condensed rDNA knob is a part of the locus containing a subset of inactive rRNA genes silenced by heterochromatinization and that the dispersed rDNA chromatin fraction is a domain containing a subset of transcriptionally active rRNA genes.

According to the presence of epigenetic marks, silver staining and deposits of reduced DAB, it can be concluded that active rRNA copies reside within the dispersed rDNA chromatin fraction of the NOR-1 locus in root tip cycling cells of the common oak. Interestingly, even though the condensed rDNA knob of the NOR-1 locuswas enriched with the repressive histone mark H3K9me1 and poor in the active histone mark H3K4me3, it showed a low level of 5-mC and a mild co-localization with the H3K9ac mark, both hallmarks of permissive chromatin. In addition, the rDNA knob showed mild silver precipitation. One plausible explanation for this would be that rRNA genes within the rDNA knob of NOR-1 locus stay hypomethylated to be accessible for rapid reactivation, which can be achieved by a change in histone modifications, mostly demethylation of Lys 9 of histone H3 and acetylation at the same position. Cytosine methylation is a more stable epigenetic modification than histone marks and gradually and slowly changes on a larger scale, i.e. along a longer chromosome region occupied by rRNA genes [54]. Another possibility would be that within the rDNA knob of NOR-1 locus, copies of silent and active rRNA genes are intermingled. Indeed, ChIP analysis has demonstrated that in the hybrid *A. suecica*, which shows nuclear dominance, the dominant *A. arenosa* rRNA genes associate with both active and silent histone marks. The same situation is found in non-hybrid *A. thaliana* in which a fraction of rRNA genes within the same locus associates with H3K4me3 and another fraction associates with H3K9me2 [5].

The dispersed rDNA chromatin fraction of NOR-1 locus comprised smaller peri- and intra-nucleolar rDNA chromatin knobs (FISH dots), as visible in LM, interconnected with thin threads of chromatin suggesting that both transcriptionally silent and active rRNA genes are possibly present in this fraction. ISH using 18S probes on ultra-thin sections of oak root tips revealed condensed sites on the nucleoplasm-nucleolar boundary that sometimes protruded into the nucleolar mass while decondensed rDNA chromatin was clearly spread in the volume of the nucleolus. It is possible that the condensed rDNA chromatin on the nucleoplasm-nucleolar boundary represents an intermediate heterochromatin state, which under appropriate conditions might become transcriptionally active [55]–[57]. Pontvianne et al. [11] have shown that localization of rRNA copies inside or outside of the nucleolus reflects transcriptional and epigenetic states that are changeable according to the needs of the cell and they further reasoned that the excess rRNA genes coalesce into dense heterochromatic structures at the external edge of the nucleolus but spool out into the nucleolus as more rRNA genes are needed. Conversely, excess rRNA genes would be reeled into the external reservoir such that rRNA gene partitioning between the nucleolus and nucleoplasm is the cytological manifestation of dynamic rRNA gene dosage control.

Study of chromatin topology of the NOR-1 locus in different cells of *Q. robur* root tip led to a conclusion that the active fraction of rRNA genes within the NOR-1 is not fully random, as the (pro)metaphase NOR sites always show the same condensation pattern at the same position of the NOR-1 sites. In addition, stable dimorphism was observed for this locus: while dispersed rDNA chromatin fraction was located at the terminal part of one site, at another site it connected the proximal rDNA knob with the condensed rDNA chromatin on the satellite, i.e. dispersed rDNA chromatin corresponded to SC. The mechanisms underlying this stable dimorphism of NOR-1 sites, reflecting the choice of rRNA copies to be transcribed in oak root tip cycling cells, remains unknown. We hypothesize that epigenetic mechanisms responsible for cell memory are involved.

## Supporting Information

Figure S1 **Reduction of the global level of 5-mC in root tip cycling cells of** ***Q.robur*** **following treatment with 5-aza-2′-dC.** Immunolocalization of 5-mC before (A) and following the epigenetic treatment (B). C. Integrated fluorescence intensity analysis confirmed reduction of global 5-mC levels following treatment. Number of asterisks indicates p<0.0001; error bars represent the standard error of the mean. Scale bar is 5 µm.
(TIF)

Figure S2 **Examples of full Z-stacks for measurement of NOR-1 and NOR-2 size.** Size was measured only when features were fully included in the confocal image stack. Two nuclei are given (upper panel. lower panel). Green channel represents chromatin while the red channel corresponds to 18S rDNA signal. Upper and lower panel are at different scales.
(TIF)

Figure S3 **Examples of full Z-stacks for measurement of decondensation percentage.** Two nuclei are shown: one with both NOR-1 sites visible (upper panel), and another with only one NOR-1 site visible (lower panel). Green channel represents chromatin while the red channel corresponds to 18S rDNA signal. Upper and lower panel are at slightly different scales.
(TIF)

Table S1 **Measured maximum cross-section (Area) and integrated volume (Volume) for NOR-1 and NOR-2 loci.** All values are in arbitrary OD units normalized by pixel area or voxel volume to make them directly comparable across images.
(XLSX)

## References

[1] Grummt I, Pikaard CS (2003) Epigenetic silencing of RNA polymerase I transcription. Nat Rev Mol Cell Biol 4: 641–649. 12923526 10.1038/nrm1171

[2] Carmo-Fonseca M, Mendes-Soares L, Campos I (2000) To be or not to be in the nucleolus. Nat Cell Biol 2: E107–112. 10854340 10.1038/35014078

[3] Preuss SB, Costa-Nunes P, Tucker S, Pontes O, Lawrence RJ, et al (2008) Multimegabase silencing in nucleolar dominance involves siRNA-directed DNA methylation and specific methylcytosine-binding proteins. Mol Cell 32: 673–684. 19061642 10.1016/j.molcel.2008.11.009 PMC2741319

[4] Pikaard C, Pontes O (2007) Heterochromatin: condense or excise. Nat Cell Biol 9: 19–20. 17199128 10.1038/ncb0107-19

[5] Lawrence RJ, Earley K, Pontes O, Silva M, Chen ZJ, et al (2004) A concerted DNA methylation/histone methylation switch regulates rRNA gene dosage control and nucleolar dominance. Mol Cell 13: 599–609. 14992728 10.1016/s1097-2765(04)00064-4

[6] Matyasek R, Tate JA, Lim YK, Srubarova H, Koh J, et al (2007) Concerted evolution of rDNA in recently formed Tragopogon allotetraploids is typically associated with an inverse correlation between gene copy number and expression. Genetics 176: 2509–2519. 17603114 10.1534/genetics.107.072751 PMC1950650

[7] Chen ZJ, Pikaard CS (1997) Epigenetic silencing of RNA polymerase I transcription: a role for DNA methylation and histone modification in nucleolar dominance. Genes & Development 11: 2124–2136. 9284051 10.1101/gad.11.16.2124 PMC316451

[8] Preuss S, Pikaard CS (2007) rRNA gene silencing and nucleolar dominance: insights into a chromosome-scale epigenetic on/off switch. Biochim Biophys Acta 1769: 383–392. 17439825 10.1016/j.bbaexp.2007.02.005 PMC2000449

[9] Vieira R, Queiroz A, Morais L, Barão A, Mello-Sampayo T, Viegas W (1990) 1R chromosome nucleolus organizer region activation by 5-azacytidine in wheat x rye hybrids. Genome 33: 707–712.

[10] Chen ZJ, Comai L, Pikaard CS (1998) Gene dosage and stochastic effects determine the severity and direction of uniparental ribosomal RNA gene silencing (nucleolar dominance) in Arabidopsis allopolyploids. Proc Natl Acad Sci U S A 95: 14891–14896. 9843986 10.1073/pnas.95.25.14891 PMC24546

[11] Pontvianne F, Blevins T, Chandrasekhara C, Mozgova I, Hassel C, et al (2013) Subnuclear partitioning of rRNA genes between the nucleolus and nucleoplasm reflects alternative epiallelic states. Genes Dev 27: 1545–1550. 23873938 10.1101/gad.221648.113 PMC3731543

[12] Roussel P, Hernandez-Verdun D (1994) Identification of Ag-NOR proteins, markers of proliferation related to ribosomal gene activity. Exp Cell Res 214: 465–472. 7523152 10.1006/excr.1994.1283

[13] Roussel P, Andre C, Comai L, Hernandez-Verdun D (1996) The rDNA transcription machinery is assembled during mitosis in active NORs and absent in inactive NORs. J Cell Biol 133: 235–246. 8609158 10.1083/jcb.133.2.235 PMC2120807

[14] Sirri V, Roussel P, Hernandez-Verdun D (2000) The AgNOR proteins: qualitative and quantitative changes during the cell cycle. Micron 31: 121–126. 10588057 10.1016/s0968-4328(99)00068-2

[15] Rawlins DJ, Shaw PJ (1990) Localization of ribosomal and telomeric DNA sequences in intact plant nuclei by in-situ hybridization and three-dimensional optical microscopy. J Microsc 157: 83–89. 2299663 10.1111/j.1365-2818.1990.tb02949.x

[16] Leitch AR, Mosgoller W, Shi M, Heslop-Harrison JS (1992) Different patterns of rDNA organization at interphase in nuclei of wheat and rye. J Cell Sci 101 (Pt 4): 751–757. 10.1242/jcs.101.4.751 1527177

[17] Caperta AD, Neves N, Morais-Cecilio L, Malho R, Viegas W (2002) Genome restructuring in rye affects the expression, organization and disposition of homologous rDNA loci. J Cell Sci 115: 2839–2846. 12082145 10.1242/jcs.115.14.2839

[18] Pontes O, Lawrence RJ, Neves N, Silva M, Lee JH, et al (2003) Natural variation in nucleolar dominance reveals the relationship between nucleolus organizer chromatin topology and rRNA gene transcription in Arabidopsis. Proc Natl Acad Sci U S A 100: 11418–11423. 14504406 10.1073/pnas.1932522100 PMC208772

[19] Caperta AD, Neves N, Viegas W, Pikaard CS, Preuss S (2007) Relationships between transcription, silver staining, and chromatin organization of nucleolar organizers in Secale cereale. Protoplasma 232: 55–59. 18157499 10.1007/s00709-007-0277-4

[20] Silva M, Pereira HS, Bento M, Santos AP, Shaw P, et al (2008) Interplay of ribosomal DNA loci in nucleolar dominance: dominant NORs are up-regulated by chromatin dynamics in the wheat-rye system. PLoS One 3: e3824. 19048103 10.1371/journal.pone.0003824 PMC2585015

[21] Santos AP, Serra T, Figueiredo DD, Barros P, Lourenco T, et al (2011) Transcription regulation of abiotic stress responses in rice: a combined action of transcription factors and epigenetic mechanisms. OMICS 15: 839–857. 22136664 10.1089/omi.2011.0095

[22] Wallace H, Langridge WHR (1971) Differential amphiplasty and the control of ribosomal RNA synthesis. Heredity 27: 1–13.

[23] Shaw P, McKeown P (2011) The Structure of rDNA Chromatin. In: Olson MOJ, editor. The Nucleolus: Springer New York. 43–55.

[24] Heitz E (1931) Nukleolen und Chromosomen in der Gattung *Vicia* . Planta 15: 495–505.

[25] McClintock B (1934) The relationship of a particular chromosomal element to the development of the nucleoli in *Zea mays* . Zeitschrift fur Zellforschung und Mikroskopische Anatomie 21: 294–328.

[26] Melese T, Xue Z (1995) The nucleolus: an organelle formed by the act of building a ribosome. Curr Opin Cell Biol 7: 319–324. 7662360 10.1016/0955-0674(95)80085-9

[27] Gonzalez IL, Sylvester JE (1997) Beyond ribosomal DNA: on towards the telomere. Chromosoma 105: 431–437. 9211970 10.1007/BF02510479

[28] Scheer U, Hock R (1999) Structure and function of the nucleolus. Curr Opin Cell Biol 11: 385–390. 10395554 10.1016/S0955-0674(99)80054-4

[29] Dousset T, Wang C, Verheggen C, Chen D, Hernandez-Verdun D, et al (2000) Initiation of nucleolar assembly is independent of RNA polymerase I transcription. Mol Biol Cell 11: 2705–2717. 10930464 10.1091/mbc.11.8.2705 PMC14950

[30] Hernandez-Verdun D (2006) Nucleolus: from structure to dynamics. Histochem Cell Biol 125: 127–137. 16328431 10.1007/s00418-005-0046-4

[31] McKeown PC, Shaw PJ (2009) Chromatin: linking structure and function in the nucleolus. Chromosoma 118: 11–23. 18925405 10.1007/s00412-008-0184-2

[32] Raska I (2004) Searching for active ribosomal genes. Prog Mol Subcell Biol 35: 23–56. 15113078

[33] Raska I, Koberna K, Malinsky J, Fidlerova H, Masata M (2004) The nucleolus and transcription of ribosomal genes. Biol Cell 96: 579–594. 15519693 10.1016/j.biolcel.2004.04.015

[34] Kalmarova M, Smirnov E, Masata M, Koberna K, Ligasova A, et al (2007) Positioning of NORs and NOR-bearing chromosomes in relation to nucleoli. J Struct Biol 160: 49–56. 17698369 10.1016/j.jsb.2007.06.012 PMC2446407

[35] Zoldos V, Papes D, Cerbah M, Panaud O, Besendorfer V, et al (1999) Molecular-cytogenetic studies of ribosomal genes and heterochromatin reveal conserved genome organization among 11 Quercus species. Theoretical and Applied Genetics 99: 969–977.

[36] Torres-Ruiz RA, Hemleben V (1994) Pattern and degree of methylation in ribosomal RNA genes of Cucurbita pepo L. Plant Mol Biol. 26: 1167–1179. 10.1007/BF00040697 7811974

[37] Gerlach WL, Dyer TA (1980) Sequence organization of the repeating units in the nucleus of wheat which contain 5S rRNA genes. Nucleic Acids Res 8: 4851–4865. 7443527 10.1093/nar/8.21.4851 PMC324264

[38] Vičić V, Barišić D, Horvat T, Biruš I, Zoldos V (2013) Epigenetic characterization of chromatin in cycling cells of pedunculate oak, Quercus robur L. Tree Genetics & Genomes. 9: 1247–1256.

[39] Manickavelu A, Kambara K, Mishina K, Koba T (2007) An efficient method for purifying high quality RNA from wheat pistils. Colloids Surf B Biointerfaces 54: 254–258. 17142017 10.1016/j.colsurfb.2006.10.024

[40] Bolte S, Cordelieres FP (2006) A guided tour into subcellular colocalization analysis in light microscopy. J Microsc 224: 213–232. 17210054 10.1111/j.1365-2818.2006.01706.x

[41] Schneider CA, Rasband WS, Eliceiri KW (2012) NIH Image to ImageJ: 25 years of image analysis. Nat Methods 9: 671–675. 22930834 10.1038/nmeth.2089 PMC5554542

[42] R Core t (2013) A language and environment for statistical computing. Foundation for Statistical Computing.

[43] Harrell FE DC (1982) A new distribution-free quantile estimator. Biometrika 69.

[44] Harrell FE DC (2014) Hmisc: Harrell Miscellaneous. R package version 3.14–4.

[45] Davison AC HD (1997) Bootstrap Methods and Their Applications. Cambridge University Press, Cambridge.

[46] Canty A RB (2013) Bootstrap R (S-Plus) Functions. R package version 1.3–9.

[47] Bauer N, Horvat T, Birus I, Vicic V, Zoldos V (2009) Nucleotide sequence, structural organization and length heterogeneity of ribosomal DNA intergenic spacer in Quercus petraea (Matt.) Liebl. and Q. robur L. Mol Genet Genomics 281: 207–221. 19052776 10.1007/s00438-008-0404-8

[48] CLSI (2008) Defining, Establishing, and Verifying Reference Intervals in the Clinical Laboratory; Approved Guideline–Third Edition. CLSI document C28-A3. Wayne, PA: Clinical and Laboratory Standards Institute.

[49] Tucker S, Vitins A, Pikaard CS (2010) Nucleolar dominance and ribosomal RNA gene silencing. Curr Opin Cell Biol 22: 351–356. 20392622 10.1016/j.ceb.2010.03.009 PMC2912983

[50] Amado L, Abranches R, Neves N, Viegas W (1997) Development-dependent inheritance of 5-azacytidine-induced epimutations in triticale: analysis of rDNA expression patterns. Chromosome Res 5: 445–450. 9421260 10.1023/a:1018460828720

[51] Probst AV, Fagard M, Proux F, Mourrain P, Boutet S, et al (2004) Arabidopsis histone deacetylase HDA6 is required for maintenance of transcriptional gene silencing and determines nuclear organization of rDNA repeats. Plant Cell 16: 1021–1034. 15037732 10.1105/tpc.018754 PMC412874

[52] Muir G, Fleming CC, Schlotterer C (2001) Three divergent rDNA clusters predate the species divergence in Quercus petraea (Matt.) Liebl. and Quercus robur L. Mol Biol Evol 18: 112–119. 11158370 10.1093/oxfordjournals.molbev.a003785

[53] Modrich P, Lahue R (1996) Mismatch repair in replication fidelity, genetic recombination, and cancer biology. Annu Rev Biochem 65: 101–133. 8811176 10.1146/annurev.bi.65.070196.000533

[54] Frigola J, Song J, Stirzaker C, Hinshelwood RA, Peinado MA, et al (2006) Epigenetic remodeling in colorectal cancer results in coordinate gene suppression across an entire chromosome band. Nat Genet 38: 540–549. 16642018 10.1038/ng1781

[55] Jenuwein T, Allis CD (2001) Translating the histone code. Science 293: 1074–1080. 11498575 10.1126/science.1063127

[56] Habu Y, Mathieu O, Tariq M, Probst AV, Smathajitt C, et al (2006) Epigenetic regulation of transcription in intermediate heterochromatin. EMBO Rep 7: 1279–1284. 17082818 10.1038/sj.embor.7400835 PMC1794695

[57] Veiseth SV, Rahman MA, Yap KL, Fischer A, Egge-Jacobsen W, et al (2011) The SUVR4 histone lysine methyltransferase binds ubiquitin and converts H3K9me1 to H3K9me3 on transposon chromatin in Arabidopsis. PLoS Genet 7: e1001325. 21423664 10.1371/journal.pgen.1001325 PMC3053343

