# Supplementary figures and images for: Inhibition of DNA Methylation Alters Chromatin Organization, Nuclear Positioning and Activity of 45S rDNA Loci in Cycling Cells of Q. robur
Source: PLoS One. 2014 Aug 5;9(8):e103954. doi: 10.1371/journal.pone.0103954 (PMC4122370; doi:10.1371/journal.pone.0103954)

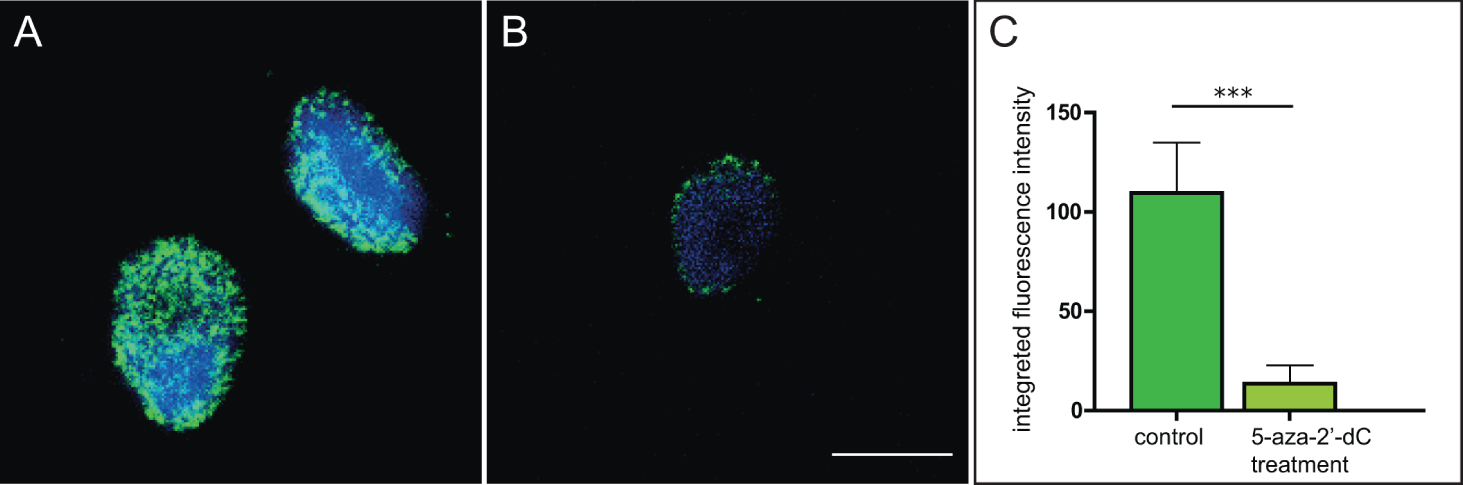

Supplement: Figure S1 — Reduction of the global level of 5-mC in root tip cycling cells of Q.robur following treatment with 5-aza-2′-dC. Immunolocalization of 5-mC before (A) and following the epigenetic treatment (B). C. Integrated fluorescence intensity analysis confirmed reduction of global 5-mC levels following treatment. Number of asterisks indicates p<0.0001; error bars represent the standard error of the mean. Scale bar is 5 µm. (TIF) [file pone.0103954.s001.tif]

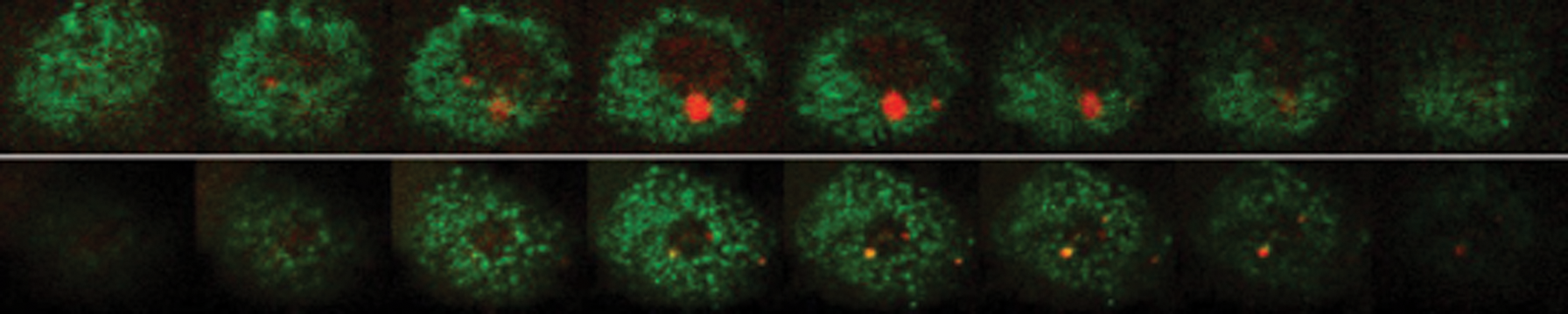

Supplement: Figure S2 — Examples of full Z-stacks for measurement of NOR-1 and NOR-2 size. Size was measured only when features were fully included in the confocal image stack. Two nuclei are given (upper panel. lower panel). Green channel represents chromatin while the red channel corresponds to 18S rDNA signal. Upper and lower panel are at different scales. (TIF) [file pone.0103954.s002.tif]

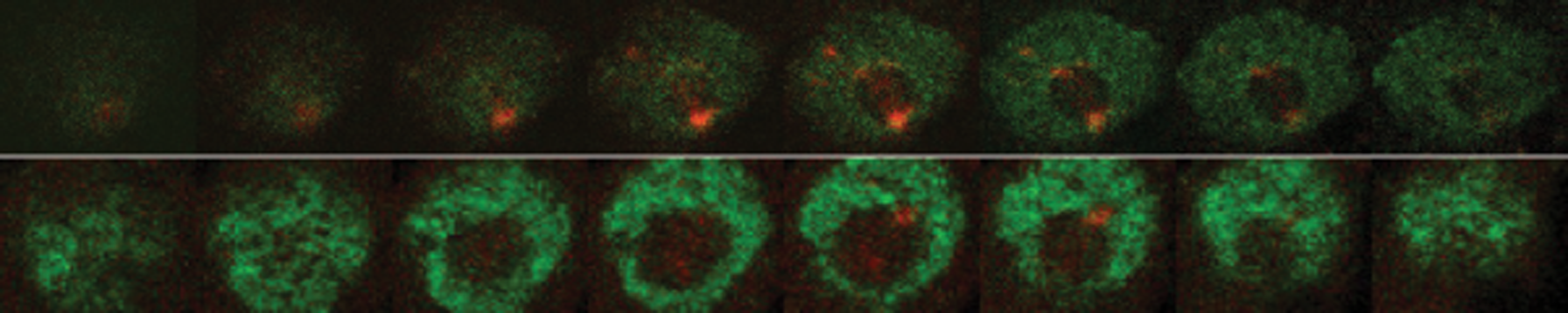

Supplement: Figure S3 — Examples of full Z-stacks for measurement of decondensation percentage. Two nuclei are shown: one with both NOR-1 sites visible (upper panel), and another with only one NOR-1 site visible (lower panel). Green channel represents chromatin while the red channel corresponds to 18S rDNA signal. Upper and lower panel are at slightly different scales. (TIF) [file pone.0103954.s003.tif]
